# Genomic and functional analysis of phage‐mediated horizontal gene transfer in *Pseudomonas syringae* on the plant surface

**DOI:** 10.1111/nph.18573

**Published:** 2022-12-02

**Authors:** Michelle T. Hulin, Mojgan Rabiey, Ziyue Zeng, Andrea Vadillo Dieguez, Sophia Bellamy, Phoebe Swift, John W. Mansfield, Robert W. Jackson, Richard J. Harrison

**Affiliations:** ^1^ NIAB Lawrence Weaver Road Cambridge CB3 0LE UK; ^2^ The Sainsbury Laboratory Norwich NR4 7UH UK; ^3^ School of Biosciences and the Birmingham Institute of Forest Research University of Birmingham Birmingham B15 2TT UK; ^4^ Faculty of Natural Sciences Imperial College London London SW7 2BX UK; ^5^ Present address: Plant Science Group Wageningen University and Research Wageningen 6708WB the Netherlands

**Keywords:** bacterial evolution, genomic diversity, horizontal gene transfer (HGT), phyllosphere, prophage, *Pseudomonas syringae*

## Abstract

- Many strains of *Pseudomonas* colonise plant surfaces, including the cherry canker pathogens, *Pseudomonas syringae* pathovars *syringae* and *morsprunorum.* We have examined the genomic diversity of *P. syringae* in the cherry phyllosphere and focused on the role of prophages in transfer of genes encoding Type 3 secreted effector (T3SE) proteins contributing to the evolution of virulence.
- Phylogenomic analysis was carried out on epiphytic pseudomonads in the UK orchards. Significant differences in epiphytic populations occurred between regions. Nonpathogenic strains were found to contain reservoirs of T3SE genes. Members of *P. syringae* phylogroups 4 and 10 were identified for the first time from *Prunus*.
- Using bioinformatics, we explored the presence of the gene encoding T3SE HopAR1 within related prophage sequences in diverse *P. syringae* strains including cherry epiphytes and pathogens. Results indicated that horizontal gene transfer (HGT) of this effector between phylogroups may have involved phage. Prophages containing *hopAR1* were demonstrated to excise, circularise and transfer the gene on the leaf surface.
- The phyllosphere provides a dynamic environment for prophage‐mediated gene exchange and the potential for the emergence of new more virulent pathotypes. Our results suggest that genome‐based epidemiological surveillance of environmental populations will allow the timely application of control measures to prevent damaging diseases.

## Introduction

The survival of bacterial plant pathogens outside their hosts is well documented (Mohr *et al*., 2008; Vouga & Greub, 2016). Pathogens in the environment represent a reservoir of strains from which new outbreaks of disease may emerge. There is a need for further understanding of not only the ecology of known pathogens, but also their close relatives, harbouring genes encoding virulence determinants that may be exchanged by horizontal gene transfer (HGT). Here, we address this, focusing on the presence of the plant pathogen *Pseudomonas syringae* on the surface of cherry trees.

*Pseudomonas syringae* is a bacterial species complex that causes plant diseases in economically important crops and environmentally significant species. An example of recent devastating disease outbreaks includes bacterial canker of kiwi fruit caused by *P. syringae* pathovar (pv) *actinidiae* (McCann *et al*., 2017). The pathovar designation is based on the strain's host of isolation and ability to cause symptoms on host plants (Sarkar *et al*., 2006). The impact of genomics has, however, altered our perception of the genetically diverse *P. syringae* complex with 13 phylogroups and 19 phylogenomic species now identified (Gomila *et al*., 2017). Members of phylogroup 2 (designated *P. s*. pv *syringae*) have a wide range of source hosts, whereas those in groups 1, 3 and 4 display more host specificity. Due to this complexity, *P. syringae* remains a major threat to crop production because of inability to detect specific strains and to breed appropriate resistance (Mansfield *et al*., 2012). Bacterial canker of cherry is caused by members of six different clades of *P. syringae* but mainly by three pathovars, *P. s*. pv *morsprunorum* races 1 and 2 (*Psm* R1 and R2) and certain strains of *P. syringae* pv s*yringae* (*Pss*), which are members of phylogroups 3, 1 and 2 of *P. syringae*, respectively (Hulin *et al*., 2020). These clades have convergently evolved to cause disease on this host. The emergence of damaging outbreaks of diseases caused by *P. syringae* is being increasingly linked to the presence of the pathogens outside their host plants and the evolution of new genotypes (Lindow & Brandl, 2003; Morris *et al*., 2013).

The life cycle of cherry canker pathogens includes a phase of epiphytic colonisation of leaves and other shoot surfaces, the phyllosphere (Crosse & Garrett, 1966; Hulin *et al*., 2020). Recent advances in our knowledge of leaf microbiomes have demonstrated the diversity of bacteria adapted for survival on, as well as within, leaf tissues (Vorholt, 2012; Helmann *et al*., 2019). Strains of *Pseudomonas* that are not recognised pathogens have long been recorded as efficient colonisers of leaf surfaces, where isolates of *P. syringae* are particularly common (Hirano & Upper, 2000). The plant surface also provides a niche within which there is considerable potential for HGT between bacteria.

The genomic diversity of strains of *P. syringae* that cause the canker disease is intriguing because of apparent differences in virulence gene arsenals. Despite their assignment to different *P. syringae* phylogroups, the cherry pathogens are enriched in a common set of genes encoding effector proteins. Effectors, injected into host cells through the Type 3 secretion system (T3SS), are now recognised to be the key pathogenicity determinants of many bacterial species towards animals and plants and have a general role in the suppression of immunity (Boyd *et al*., 2012; Baltrus *et al*., 2017; Xin *et al*., 2018). In the case of cherry‐pathogenic *P. syringae* pathovars, Hulin *et al*. (2018a) used comparative genomics to find that the effector genes *hopAR1*, *hopBB1*, *hopBF1* and *hopH1* are in strains within different clades of *P. syringae* and are associated with pathogenicity in *Prunus* spp. Bioinformatics analysis also showed that these virulence Type 3 secreted effectors (T3SEs) were probably gained through HGT in the cherry‐pathogenic *P. syringae* and are associated with mobile genetic elements (Hulin *et al*., 2020). For example, alleles of *hopAR1* are found in a genomic island in strains of *Pss* and in distinct prophage sequences in *Psm* R1 and R2. The most closely related phages are the members of the Siphoviridae and Myoviridae families (Hulin *et al*., 2020). Several members of the Siphoviridae have been found with the capability of gene transfer, notably phage lambda (Palva *et al*., 1987; Ellard *et al*., 1989; Boyd *et al*., 2012).

Various studies on bacterial diseases of animals have shown that the introduction of phages which encode virulence factors can transform a nonpathogenic bacterial host into a highly pathogenic bacterium. Numerous genes encoding T3SEs have also been found within prophages, for example in *Salmonella enterica* (Brüssow *et al*., 2004; Penadés *et al*., 2015) and more recently *Citrobacter rodentium* (Magaziner *et al*., 2019). Mirold *et al*. (2001) provided proof that the T3SE‐encoding gene *sopE* was transferred through lysogenic conversion of *Salmonella typhimurium* by a lambdoid P2‐like phage. Lambdoid phages in *Escherichia coli* O157 have now been shown to harbour up to eight T3SEs (Boyd *et al*., 2012). The functionality of prophages in O157 has been examined by Asadulghani *et al*. (2009). They found that many of the prophages were inducible, released from O157 cells and transferred to other *E. coli* strains. Numerous prophages carrying virulence factors including genes for T3SEs have recently been identified in the plant pathogenic bacterium *Ralstonia solanacearum* (Greenrod *et al*., 2021), but phage‐mediated HGT has not been functionally demonstrated for a phytopathogen.

Here, we test the following hypotheses that:

1. The cherry phyllosphere harbours pathogenic and nonpathogenic strains of *P. syringae*, and both groups carry genes encoding T3SEs and toxins.
2. The composition of the epiphytic populations varies between regions in the UK.
3. Prophages containing *hopAR1* within *P. syringae* genomes excise and transfer the virulence factor between strains on the leaf surface.

Our results demonstrate the complexity of *P. syringae* populations in the cherry phylloplane and confirm that the plant surface provides a niche for the evolution of new strains of cherry canker pathogens through HGT.

## Materials and Methods

### Bacteria, plasmids and primers

Strains used in this study are listed in Table 1, plasmids in Supporting Information Table S1 and primers in Table S2. Kings Medium B (KB; King *et al*., 1954) and Lysogeny Broth (LB) or agar (Sambrook *et al*., 1989) were used to culture bacteria. Antibiotics were used at the following concentrations: kanamycin (50 μg ml^−1^), gentamycin (25 μg ml^−1^), rifampicin (100 μg ml^−1^), nitrofurantoin (100 μg ml^−1^), tetracycline (12.5 μg ml^−1^), cycloheximide (100 mg l^−1^) and cephalexin (40 mg l^−1^).

**Table 1:** Main *Pseudomonas syringae* strains used in this study.

Strain	Phylogroup	Host	Source
*Psm* R1‐5244	3	*Prunus avium* L.	Hulin *et al*. (2018b)
*Pss*‐9644	2	*Prunus avium* L.	Hulin *et al*. (2018b)
*Psm* R1‐5300	3	*Prunus domestica* L.	Hulin *et al*. (2018b)
*Psm* R2‐leaf	1	*Prunus avium* L.	Hulin *et al*. (2018b)
1‐1 E	7	*Prunus avium* L.	This study
1‐10F	4	*Prunus avium* L.	This study
1‐11C	2b	*Prunus avium* L.	This study
1‐12B	4	*Prunus avium* L.	This study
1‐12H	7	*Prunus avium* L.	This study
2‐11B	10	*Prunus avium* L.	This study
2‐11D	10	*Prunus avium* L.	This study
2‐9 E	10	*Prunus avium* L.	This study
3‐7F	10	*Prunus avium* L.	This study
4‐2H	2d	*Prunus avium* L.	This study
5‐3F	2b	*Prunus avium* L.	This study
5‐6A	1a	*Prunus avium* L.	This study
6‐3A	7	*Prunus avium* L.	This study
6‐2G	2c	*Prunus avium* L.	This study

### Sampling

Epiphytic pseudomonads were collected in May 2017 in the Southeast (Kent), Southwest (Somerset and Dorset) and West Midlands (Staffordshire and Herefordshire), UK. The sampling strategy is represented in Fig. S1. Within each region, three orchards growing four cherry (*Prunus avium* L.) varieties (Kordia, Lapins, Sweetheart and Penny) were selected. Seven trees of each cultivar were randomly selected, and three leaves and bark strips were sampled from symptomless 2‐yr‐old wood (see Tables S3, S4).

Four discs were collected per leaf using the lid of an Eppendorf tube. Stem tissue was excised using a sterile scalpel to remove a 1‐cm strip from the top layer of bark. Samples were stored in 2‐ml tubes and refrigerated before processing within 2 d by washing them in 500 μl of phosphate buffer (Renick *et al*., 2008) and shaken at 150 rpm for 1 h. Bacterial samples were concentrated by centrifugation (3700 ***g*** for 10 min), and the pellet was plated onto KBA. Two colonies with *Pseudomonas*‐like morphology (flat colonies with irregular margins) were selected and grown up in LB in a 96‐well format and stored at −80°C in 40% glycerol.

In total, 2712 samples were collected, from which 1502 bacteria were recovered. A subset with representation from each region/orchard/variety/tissue combination was generated by randomly selecting four trees per cultivar per orchard, and two strains per tree were selected at random with one from each tissue if possible. This filtering process reduced the strains down to 260, which were then screened using *Pseudomonas* genus‐specific polymerase chain reaction (PCR; Spilker *et al*., 2004), and 166 positive samples were genome‐sequenced (see Tables S3, S4 for details).

### Sequencing

DNA was extracted using CTAB (William *et al*., 2012), and sequencing libraries were prepared using the MiSeq Nextera XT (Illumina, San Diego, CA, USA) library preparation protocol with a 10 μl total volume using the Mosquito liquid handler (SPT LabTech, Melbourn, UK). One microlitre of genomic DNA (0.2–0.3 ng μl^−1^) was used as input to the library preparation. Libraries were quality‐checked using the TapeStation D1000 High Sensitivity Kit (Agilent, Santa Clara, CA, USA) to ensure the correct size range and adapter removal before sequencing with Illumina MiSeq V2 (500 cycles). MinION sequencing (Oxford Nanopore, Oxford, UK) of strains PA‐1‐10F and PA‐1‐12B was performed with kit SQK RBK004 on a FLO‐MIN‐106 (ID FAL28332).

### Bioinformatics

Illumina‐sequenced genomes were assembled using spades v.3.13 (Bankevich *et al*., 2012) on sequencing reads after trimming with fastp (Chen *et al*., 2018). Genome assemblies were then quality‐checked using Quast (Gurevich *et al*., 2013) and CheckM (Parks *et al*., 2015). MinION genomes were assembled using Unicycler v.0.4.8 (Wick *et al*., 2017) with default settings. Genomes were deposited on NCBI under BioProject PRJNA587608 (accession numbers are listed in Table S5).

Additionally, 429 *P. syringae* genomes were downloaded from NCBI in September 2018. Genomes were initially filtered based on CheckM results (≥ 95% complete and ≤ 5% contamination) and an N50 ≥ 40 000 bp. pyani (Pritchard *et al*., 2016) was used for clustering into groups with members sharing ≥ 99.95% average nucleotide identity. One representative assembly was used per group, with more complete genomes (those with a lower number of contigs) prioritised. Two hundred thirty‐four genomes remained after this filtering. The genome of *P. putida* KT2440 was also downloaded to use as an out‐group in phylogenetic analysis. Prokka (Seemann, 2014) was utilised for annotation and orthoFinder (Emms & Kelly, 2019) to cluster proteins by orthology. Assembly details are given in Table S5.

Newly sequenced strains and the other *Pseudomonas* genomes were clustered into phylogenetic groups with ≥ 95% average nucleotide identity, which loosely correspond to ‘genomospecies’. To understand how the different isolation variables dictated what ANI groups were present, the frequencies of these clusters were compared using Fisher's exact test and visualised with balloon plots generated in the R software gplots package (Warnes *et al*., 2016).

### Phylogenetics

An initial multilocus sequence typing (MLST) tree was generated using sequences of housekeeping genes *acnB*, *gltA*, *gyrB*, *pgi*, *rpoD*, *gapA* and *fruK* (Marcelletti *et al*., 2011) that were extracted from each genome using Blastn, with query sequences from *P. syringae* pv tomato DC3000. The genes were aligned with clustalw (Thompson *et al*., 1994) and trimmed with gblocks (Castresana, 2000). Two genes, *fruK* and *gapA*, were excluded because alignment trimming led to the removal of sequence for some strains. The sequences for five genes were concatenated to generate a 2807‐bp alignment that was used to build a phylogeny using IQ‐Tree (Nguyen *et al*., 2014) with the model GTR + I + G and 1000 bootstraps.

A core genome phylogeny was generated for strains identified to be within phylogroups of *P. syringae* using 465 single‐copy proteins present in all strains identified with orthoFinder. Protein sequences were aligned with clustalw, trimmed with gblocks and then concatenated to generate a 65 265 amino acid alignment. This alignment was used to build a phylogeny using IQ‐Tree with the model JTT + I + G with 1000 bootstraps. A maximum‐likelihood phylogeny was also created using IQ‐Tree for the HopAR1 protein from 84 full‐length alleles using the model JTT + DCMUT + G4.

### Effector identification

The presence of the canonical T3SS and T3SEs was identified in each genome using tBlastn. The T3SS filtering was based on ≥80% of genes being present with ≥ 50% query length covered and ≥ 50% amino acid identity, as in Hulin *et al*. (2020). Identification of T3SEs was as in Hulin *et al*. (2018a) using a filtering threshold of ≥ 70% identity and ≥ 40% query length for each effector family. The total number of known effector alleles in each strain (not counting multiple copies of individual effector alleles or those that span a contig break) was predicted. The syringomycin, syringolin A and syringopeptin toxin biosynthesis gene clusters were also identified with hits ≥ 50% query length covered and ≥ 50% amino acid identity deemed to possess each cluster.

### Prophage identification and activity

Regions of 100 kb were extracted from around *hopAR1* using the coordinates generated from a tBlastn search and python scripting to extract the genomic region. Those genomes in which the gene was present, but the region around *hopAR1* was < 5 kb due to a small contig size, were classed as ‘too short’ as potentially false negatives. Prophages were identified using Prophage Hunter (Song *et al*., 2019) and Phaster (Arndt *et al*., 2016).

Excision of prophages carrying *hopAR1* in *Psm* R1‐5244, *Psm* R1‐5300, *Ps* 1‐10F and *Ps* 1‐12B after culture in KB was followed by PCR amplification of bacterial DNA including the left and right phage attachment sites (*attL* and *attR*) that demarcate phage excision. Circularised prophage was detected using F and R primers (Table S2). Polymerase chain reaction was performed using a 1‐μl DNA template with GoTaq^®^ Green Mastermix (Promega) using manufacturer's instructions.

### Prophage induction, purification and DNA extraction

Prophage induction was based on Raya & H'Bert (2009) after the promotion of excision by the treatment of bacteria with mitomycin C (MMC; Sigma‐Aldrich) and chloroform (AnalaR; VWR, Lutterworth, UK), or ultraviolet (UV) radiation (253.7 nm, 94 V, 43 mA). Briefly, an overnight culture in KB (2 × 10^8^ CFU ml^−1^) was diluted 10‐fold in a total of 10 ml and incubated for 1 h at 27°C, 200 rpm. Bacterial cells were then treated with MMC (1 μg ml^−1^) and returned to incubate for 3 h. Chloroform (1%, v/v) was added to the cells that were left to incubate for another 3 h. For the second method, after the initial 1 h, the 10 ml of culture was transferred to a sterilised glass Petri dish and irradiated with UV for 60 s. The culture was then transferred to a 50‐ml Falcon tube and shaken for 6 h. For the third method, after incubation for 4 h, bacteria were centrifuged at 3700 ***g*** for 10 min at room temperature. The pellet was resuspended in 5 ml sterile 0.1 M MgSO_4_ and UV‐irradiated as described previously. After each treatment, cells were incubated by shaking for another 3 h before centrifugation at 3700 ***g*** for 45 min at room temperature. The supernatant was removed and passed through a 0.22‐μm filter, and polyethylene glycol 8000 (Sigma‐Aldrich) was added to 8% w/v. After overnight incubation at 4°C, phages were precipitated by centrifugation (45 min, 3700 ***g*** at 4°C) and dissolved in 3 ml phage buffer (Bonilla *et al*., 2016). DNA was isolated using a Phage DNA Isolation Kit (Norgen Biotek, Thorold, ON, Canada), and PCR, as described previously, was performed to check for the presence of *hopAR1* and phage endolysin genes. To check for any chromosomal contamination, amplification of a bacterial gene (*gyrB*) was also performed.

### Insertional mutagenesis

To disrupt the phage integrase gene, *c*. 400 bp of DNA internal to the integrase (gene ID BKM19_016870) was amplified. The PCR product was gel‐purified with a Monarch DNA Gel Extraction Kit (NEB, Hitchin, UK). Fragments were ligated (T4) into the pCR2.1 cloning vector (Invitrogen Life Technologies Inc., Loughborough, UK) and transformed into OneShot^®^TOP10 competent cells (Invitrogen). Plasmid DNA was extracted using the PureLink™ Quick Plasmid Miniprep Kit (Invitrogen) and analysed by restriction mapping (EcoRI; NEB), amplification from M13 universal primers (Table S2) and sequencing (Eurofins, Ebersberg, Germany). The plasmid DNA was transformed into *Psm* R1‐5244 by electroporation. Competent cells were incubated with 10 μl of plasmid (*c*. 300 ng DNA) on ice for 30 min. Electroporation in 1‐mm cuvettes was performed (200 W, 2.0 kV and 25 μF), and cells were then suspended in 1 ml KB, incubated for 4 h at 250 rpm at 27°C and plated on KB plates containing kanamycin. Polymerase chain reaction confirmed the insertional knockout using primers from the upstream of the gene and within the insert.

### Transfer of prophage between bacteria

To facilitate detection, the prophage was marked with a gentamicin resistance (*Gm* ^*R*^) cassette. Intergenic regions of *c*. 500 bp from the prophage either side of a *Gm* ^*R*^ cassette were synthesised into the MCS region in the pTS1 vector (Twist Bioscience, San Francisco, CA, USA). The construct was transformed into NEB DH5α competent *E. coli* cells (NEB). Conjugation into *Psm* R1‐5244 was conducted via tri‐parental mating using *E. coli* DH5α harbouring the helper plasmid pRK2073. The mating mixture was plated on KBA for 24 h and then transferred to KBA supplemented with nitrofurantoin, tetracycline and gentamycin. Transconjugant *Psm* R1‐5244 colonies were negatively selected on LBA plates containing 10% sucrose. The plates were incubated overnight at 27°C, and single colonies were transferred to KBA plates with Gm and mirrored on KBA plates plus Gm and Tet. After incubation overnight at 27°C, colonies that grew only on Gm plates were tested by PCR to confirm the presence of the *Gm* ^*R*^ cassette.

Transfection of purified prophage DNA marked with the *Gm* ^*R*^ cassette into competent cells of *P. syringae* 3‐7F_Rifampicin resistant (*Rif*) was carried out by electroporation as described previously.

Movement of the prophage was examined on detached cherry leaves (from 2‐yr‐old saplings of *P. avium* L. cv Sweetheart). The surface of freshly picked leaves (1‐ to 2‐wk‐old) was sterilised with 70% ethanol and sprayed by hand with a mixture of *Psm* R1‐5244_*Gm* ^*R*^ and *Ps* 3‐7F*_Rif* resistant (OD_600_ 0.2–2 × 10^8^ CFU ml^−1^ for each strain). Controls included spraying with PBS and individual bacteria. Leaves were left to dry for 1 d to allow bacterial growth, then either left untreated or irradiated with UV for 60 s, placed in humid chambers and incubated at 22°C (16 h : 8 h, light : dark). The leaves were incubated for a maximum of 3 d. At least three leaves were inoculated for each treatment. Four discs (1 cm in diameter) were excised from each leaf and placed into a 2‐ml tube containing 1 ml of PBS with two tungsten beads and homogenized at a speed of 4 m s^−1^ for 15 s. The tubes were centrifuged briefly at 2000 ***g*** to remove plant debris, and 200 μl of supernatant was spread onto KBA plates supplemented with cycloheximide and cephalexin and either Gm, Rif or a mixture of both.

Polymerase chain reaction amplifications were performed to check for the presence of *hopAR1*, the phage endolysin gene, and *hopY1* (to confirm the identification of 3‐7F). Prophage induction tests were also carried out on recipient strains.

### Pathogenicity assay of *Pseudomonas syringae* isolates on detached cherry leaves

Detached leaf inoculation was performed following Hulin *et al*. (2018b). Freshly picked leaves (1‐ to 2‐wk‐old) were infiltrated with bacterial suspension (2 × 10^6^ CFU ml^−1^) or with 10 mM MgCl_2_ as control, from the abaxial surface using a blunt‐ended 1‐ml syringe. Leaves were then incubated as described previously before assessment. Each leaf was infiltrated four times using the same strain, and at least three leaves were inoculated with each strain. Symptom development was recorded on a 0–5 scale.

## Results

### Sampling reveals genomic diversity of epiphytic pseudomonads

**Fig. 1:**
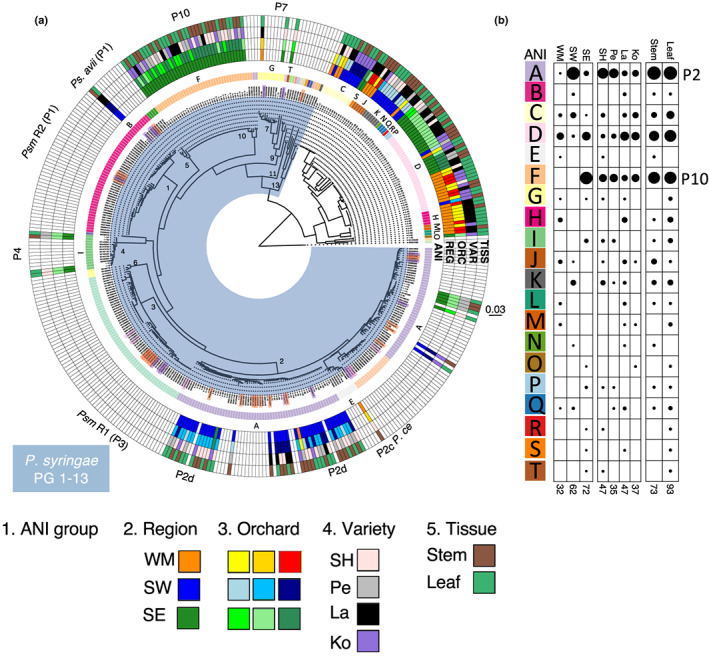
Diverse *Pseudomonas* isolates were recovered from cherry and showed distinct regional three differences. (a) A multilocus sequence typing‐based phylogenetic tree was built using five housekeeping genes (*acnB*, *gltA*, *gyrB*, *pgi* and *rpoD*) comparing isolates with genomes from NCBI. The tree was rooted to *Pseudomonas putida*, and the scale is substitutions per site. Isolates recovered from cherry in this work have the reduced naming system in the inner wheel and are defined within the rings 2–5 of the heatmap. Other *Pseudomonas* strains are labelled by assembly name, and known pathogens of stone fruits are highlighted in red (cherry), purple (other *Prunus*) and pink (other Rosaceae). The *Pseudomonas syringae* species complex phylogroups (PG) 1–13 are highlighted in blue and phylogroups (P) are noted on the dendrogram (black numerals) and outside the outer circle. The heatmap rings going outwards: (1) average nucleotide identity (ANI) 95% species clusters labelled for new isolates (used in comparative analysis in B); (2) region of isolation (WM, West Midlands; SW, Southwest; SE, Southeast); (3) orchards within these regions coloured in the same base colour as region; (4) cherry variety (SH, Sweetheart; Pe, Penny; La, Lapins; Ko, Kordia); (5) cherry tissue (green, leaf; brown, woody). (b) Balloon plot showing the frequency of each ANI species cluster dependent on region, cherry variety or tissue. Clusters are labelled from A to T corresponding to the groups in A. Dots are proportional to the number of isolates in each species group. The total number of isolates is listed at the bottom.

The cherry phyllosphere is a niche occupied by canker pathogens (Crosse & Garrett, 1966). However, little is known about the diversity and pathogenic potential of epiphytic pseudomonads on this host. To analyse the presence of *Pseudomonas* within the cherry phyllosphere, 2712 samples were collected across three regions in the UK: the Southeast, Southwest and the West Midlands. A randomised sample of 166 *Pseudomonas* strains (Tables S3, S4) was genome‐sequenced (assembly details and NCBI accession numbers are in Table S5). Phylogenetic analysis based on MLST loci (Fig. 1a) indicated that 93 of the new strains were within known *P. syringae* phylogroups (1, 2, 4, 7 and 10), whilst 73 were located outside this (Gomila *et al*., 2017). Intriguingly, none of the strains isolated from the cherry phylloplane were closely related to known pathogens *Psm* R1 and *Psm* R2 that are frequently reported as the causes of canker in the UK (labelled on Fig. 1a).

We next assessed the impact of orchard region, host variety and tissue of isolation on the genotype of *P. syringae* isolated. Strains were clustered by genome average nucleotide identity (ANI ≥ 95%) into ‘species groups’ labelled in Fig. 1(a,b). Fisher's exact test analysis revealed that region had a major influence on the *P. syringae* group isolated (*P* < 0.01), and variety was also significant (*P* < 0.01), but tissue had no major influence (*P* = 0.84). The results are visualised in balloon plots in Fig. 1(b). Two *P. syringae* phylogroups (2 and 10) accounted for 89% of all new isolates. Regional variation was striking: 51% of isolates from the Southeast belonged to phylogroup 10. By contrast, 62% of isolates from the Southwest belonged to phylogroup 2 and were closely related to *P. s*. pv *syringae* cherry pathogens. By contrast, 94% of isolates from the West Midlands belonged to diverse clades outside the known phylogroups, suggesting a greater diversity in this region.

### Reservoirs of virulence genes in epiphytes

**Fig. 2:**
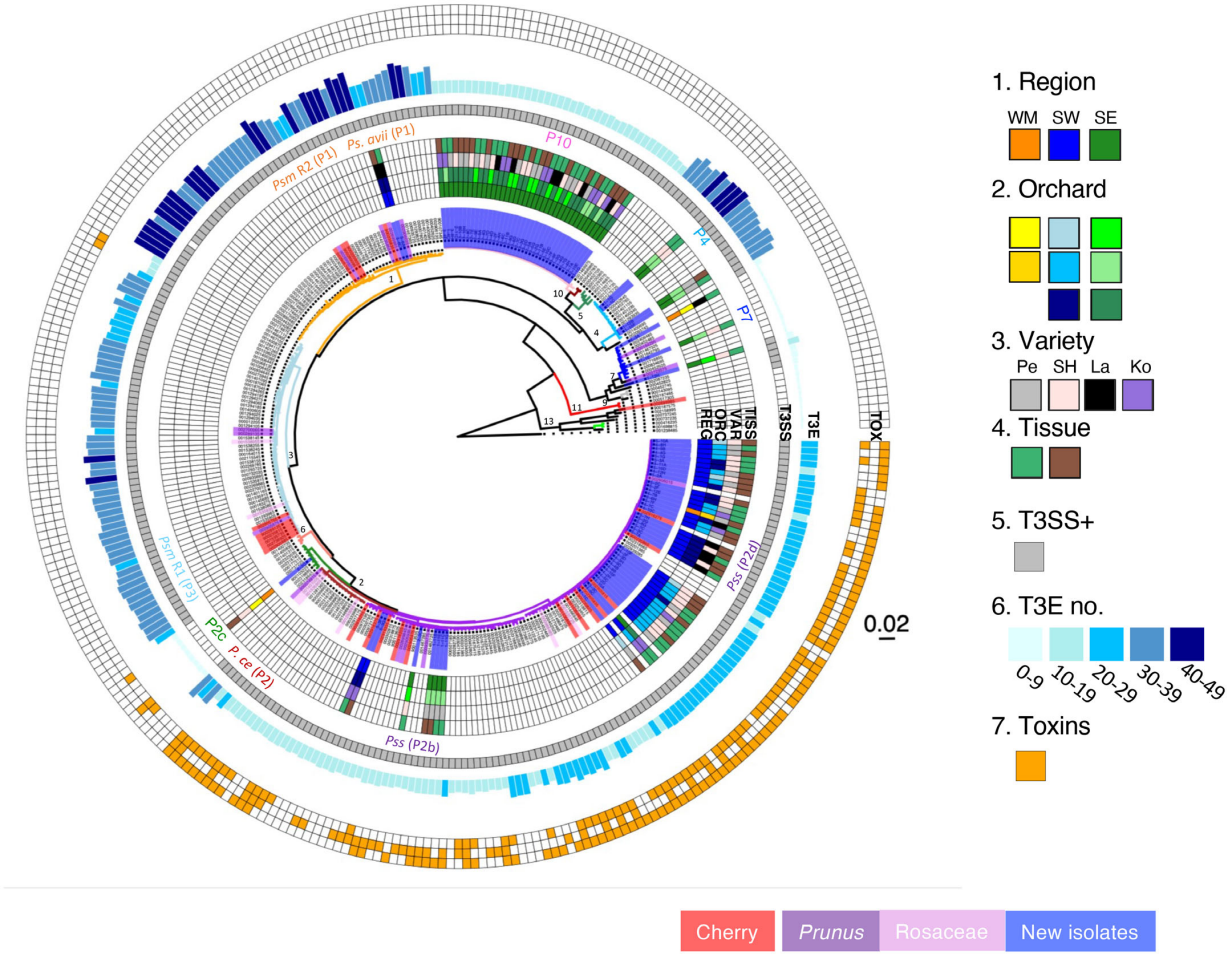
Core genome phylogenetics shows that the cherry phyllosphere supports diverse clades of *Pseudomonas syringae*, varying in Type 3 secreted effector (T3SE) and toxin repertoires. Strain identifiers (inner wheel) are highlighted if they were sampled in this study (blue), or are known pathogens of cherry (red), other *Prunus* (purple) or Rosaceae (pink), including *P. syringae* pvs *syringae* (Pss), *morsprunorum* race 1 and race 2 (*Psm* R1 and *Psm* R2), *Ps. avii* and *P. cerasi* (*P. ce*), as marked between heatmap rings 4 and 5. Phylogroups (P) are also identified between rings 4 and 5. The phylogenetic branches are coloured by average nucleotide identity (ANI) groups (≥ 95%). Heatmap going outwards: (1) region of isolation (REG); (2) orchards (ORC) within these regions coloured in the same base colour as region; (3) cherry variety (VAR) (Pe, Penny; SH, Sweetheart; La, Lapins; Ko, Kordia); (4) cherry tissue (TISS), (green, leaf; brown, woody); (5) presence of the canonical *hrp* gene‐encoded Type 3 secretion system (T3SS); (6) number of known T3SE genes; (7) presence of toxins (TOX) (from inner to outer circle: i, syringolin A; ii, syringomycin; iii, Syringopeptin). Major groups of interest are labelled on the tree with numbers referring to the phylogroup. The scale shows amino acid substitutions per site.

Our focus was on identifying *P. syringae* strains in the cherry phyllosphere that may have the potential to be canker pathogens; therefore, the 93 strains within the known species complex were analysed in further detail. A core genome phylogeny was created (Fig. 2), and the presence of genes encoding the T3SS and known T3SEs and toxins was mapped onto this phylogeny (Fig. 2). This analysis revealed that new epiphytic strains possessed repertoires of virulence factors similar to those in their close relatives within each phylogroup. For example, phylogroup 10 isolates had a low number of T3SEs (14–16), whereas phylogroups 1 and 4 strains had a high number (28 in phylogroup 1 strains and between 37 and 41 in phylogroup 4). An intermediate number of effectors (17–22 per genome) and up to three toxin biosynthetic gene clusters were characteristically found in phylogroup 2 strains. Finally, phylogroup 7 strains did not have a complete T3SS and few predicted T3SEs. The complete presence/absence data for all effectors and toxins are presented in Table S6.

**Fig. 3:**
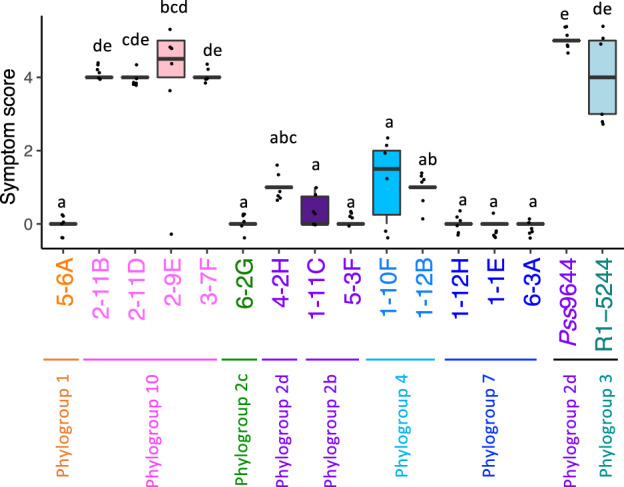
Pathogenicity test of 14 representative isolates on cherry leaves. Cherry leaves were infiltrated with each isolate, and symptoms were scored after 7 d. Strain identity and phylogroup are shown. Cherry pathogens *Pss*9644 and *Psm* R1‐5244 were included for comparison. Symptom scoring was as in Hulin *et al*. (2018b). 0, no symptoms; 1, isolated flecking; 2, browning of < 50% of inoculation site; 3, browning > 50% of the site; 4, 100% browning; 5, 100% browning and spread from the zone of infiltration. Mock inoculation with 10 mM MgCl_2_ caused no symptom development. Strains are coloured based on their *Pseudomonas syringae* phylogroup as recorded on the *x*‐axis. The box plots created in R (ggplot2) show median, 25^th^ and 75^th^ percentiles and outliers. Statistical groupings after ANOVA analysis are highlighted with different letters. This experiment was performed twice, and both datasets were combined and presented here.

Pathogenicity on cherry was tested for 14 epiphytes representative of each of the different phylogroups as shown in Fig. 2. Strains were inoculated into detached leaves and symptoms scored after 7 d (Fig. 3). Interestingly, new isolates from phylogroup 10 were able to cause disease lesions comparable to those caused by cherry pathogens *Psm* R1 and *Pss*, meaning that the new group, which has not been isolated from cherry before, may have pathogenic potential on cherry. Isolates from the other phylogroups 1, 2, 4 and 7 were at best only weakly virulent causing limited or no symptoms. This indicated that low virulence *P. syringae* strains possessing up to 41 T3SE‐encoding genes and sometimes additional toxin gene clusters colonise the cherry phyllosphere but are not pathogenic to this host. Shuffling of their T3SE repertoire through gains and losses could lead to the emergence of pathogenic lineages.

### The *hopAR1* effector gene is located within homologous prophage sequences in epiphytes and pathogens

**Fig. 4:**
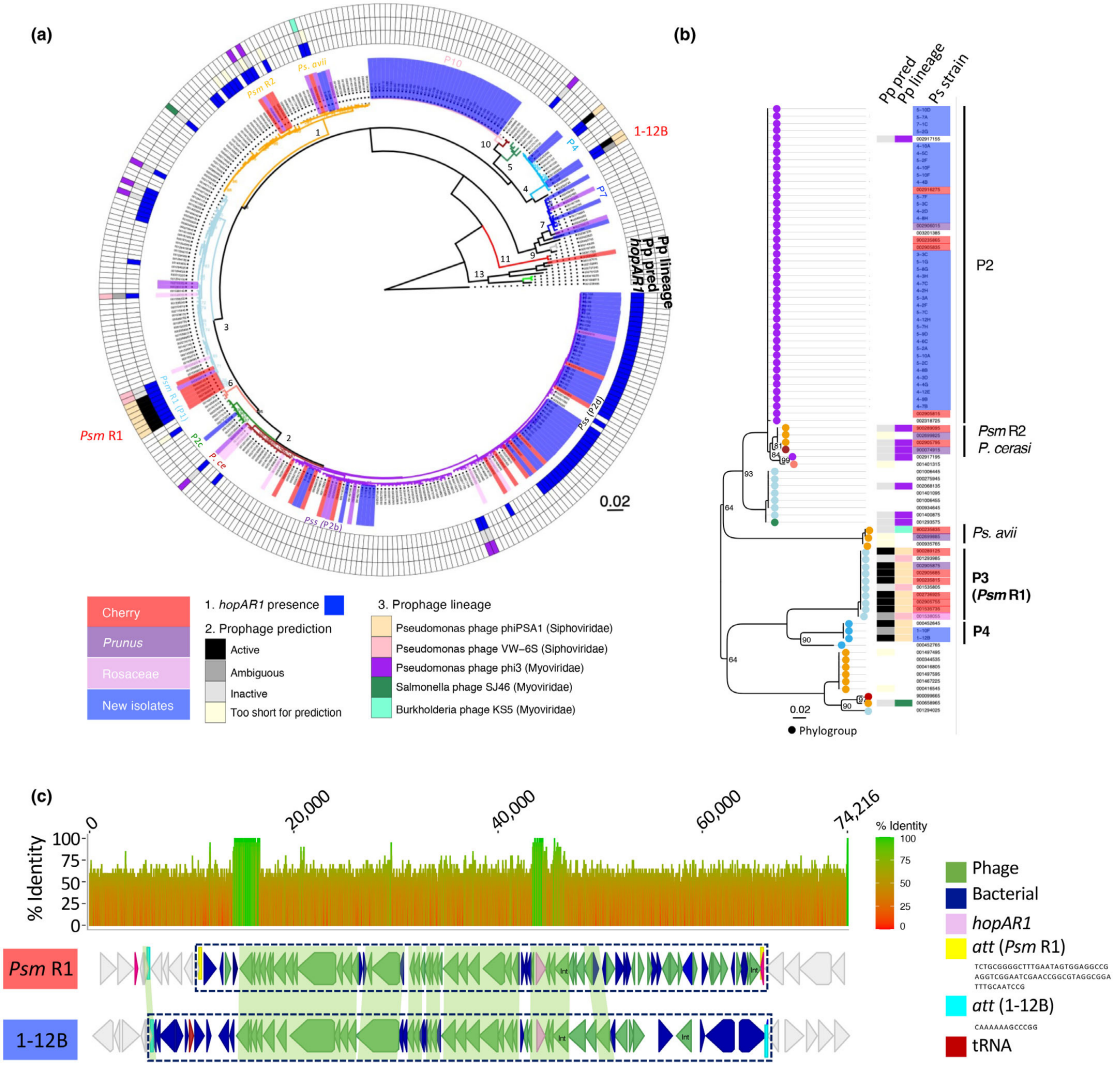
*hopAR1* gene is located in prophage sequences common to known cherry pathogens and new isolates from the phyllosphere. (a) Presence of *hopAR1* annotated onto the core genome phylogeny as displayed in Fig. 2 showing phylogroups (P) labelled on the tree and the identity of strains, from the cherry phyllosphere (light blue), known pathogens of cherry (red), other *Prunus* (purple) and Rosaceae (pink). The three heatmap circles going outwards record: 1, *hopAR1* gene presence (blue); 2, presence of prophage (Pp) sequences surrounding *hopAR1* and if these prophages were predicted to be active, ambiguous, inactive or the region was too short for accurate prediction; 3, the most homologous phage taxon predicted by Prophage Hunter. (b) A maximum‐likelihood phylogeny of the HopAR1 protein from 84 strains with full‐length HopAR1 alleles. Strains are highlighted as in (a). Circles at each tip are coloured based on the phylogroup of each strain as in phylogeny in (a). Bootstrap support values are labelled at inner nodes where they are below 100. Phylogroups (P) and cherry pathogen lineages are labelled. (c) Alignment of the 74 216 bp region including the *hopAR1* gene in Psm R1‐5244 and 1‐12B. Similarity between the two sequences is shown on the above plot of nucleotide identity over sliding windows of 20 nucleotides. The plot is coloured by a scale from red (low) to green (high) identity ranging from 0% to 100%. The prophage region is within the dashed rectangle. The key denotes whether genes were annotated as phage or bacterial by Phaster and other key regions including the predicted attachment (*att*) sites. Int, integrase.

Our previous work revealed that distantly related cherry pathogens commonly share genes encoding T3SEs on mobile genetic elements. One such gene, *hopAR1*, is present in most cherry pathogens and is located within divergent prophage sequences in *Psm* R1, *Psm* R2 and *Pseudomonas cerasi* (Hulin *et al*., 2020). As the cherry phyllosphere might be an environment enabling prophage movement between diverse *P. syringae* lineages, we screened the epiphytic strains for the presence of this effector and found that 41 of the 93 new *P. syringae* isolates from the cherry phyllosphere possessed *hopAR1* (Fig. 4a). Most strains in phylogroup 2 had the gene, as was expected due to their close relatedness to *Pss* cherry pathogens that also possess *hopAR1*. Two new isolates from within phylogroup 4 (1‐10F and 1‐12B) also possessed *hopAR1*. Interestingly, the other two strains within phylogroup 4 (1‐1C and 1‐1G) did not harbour *hopAR1*, even though they were isolated from the same orchard, but from a different cherry variety. Closely related strains may therefore vary in effector repertoires even within the same orchard.

Because of the common association of *hopAR1* with prophages in cherry pathogens, we determined whether any of the new isolates also harbour the gene within a prophage. We scanned all 327 *P. syringae* genomes used in the core genome phylogeny for *hopAR1* and used its location to extract the surrounding regions, which were analysed by Prophage Hunter and Phaster (see Table S7). The *hopAR1* effector genes of phylogroup 4 strains 1‐10F and 1‐12B, as well as several other strains within this phylogroup, were located within prophage sequences, indicating the gene may have been gained in these strains via phage transfer. The prophages within 1‐10F and 1‐12B were closely homologous to the phage in the phylogroup 3 strain *Psm* R1 (the closest phage was *Pseudomonas* Phage phiPSA, a member of the Siphoviridae), indicating that related phage lineages carry this effector in distantly related phylogroups. To test this hypothesis, a phylogeny based on the HopAR1 protein sequences was constructed (Fig. 4b). This showed that the HopAR1 sequences of the two new isolates and other phylogroup 4 members were close out‐groups to the *Psm* R1 HopAR1 protein sharing >80% sequence identity, supporting past transfer of this effector between phylogroups within the Siphoviridae prophages.

The two phylogroup 4 strains were resequenced using the Oxford Nanopore MinION to generate complete genomes. The whole phage region was identified in the genome of phylogroup 4 strain 1‐12B and aligned to the prophage in *Psm* R1 (Fig. 4c). The prophage carrying *hopAR1* in 1‐12B and Psm R1‐5244 were predicted to be active (scoring 0.8 and 0.9 on Prophage Hunter). Overall, there was a high degree of synteny in the first half of the two prophages, including core phage genes such as the tail‐shaft, tail‐fibre and coat‐encoding genes, the region containing *hopAR1* and a downstream integrase. However, downstream regions varied significantly, indicating some possible degradation of the phage and/or import of new sequences. The attachment (*att*) sites were also predicted to differ between 1‐12B and *Psm* R1. The *Psm* R1 *att* site was much longer (67 bp compared with 13 bp) and within a tRNA gene. The 1‐12B *attL* sequence was present in *Psm* R1, but there was no corresponding *attR*.

Further alignments of this region in the other phylogroup 4 strain 1‐10F and some other phylogroup 4 genomes with the *hopAR1* prophage revealed substantial variation in the second half of the prophage indicating possible recombination events leading to loss or gain of new sequences (Fig. S2).

### The prophage excised from the bacterial host chromosome and circularised

**Fig. 5:**
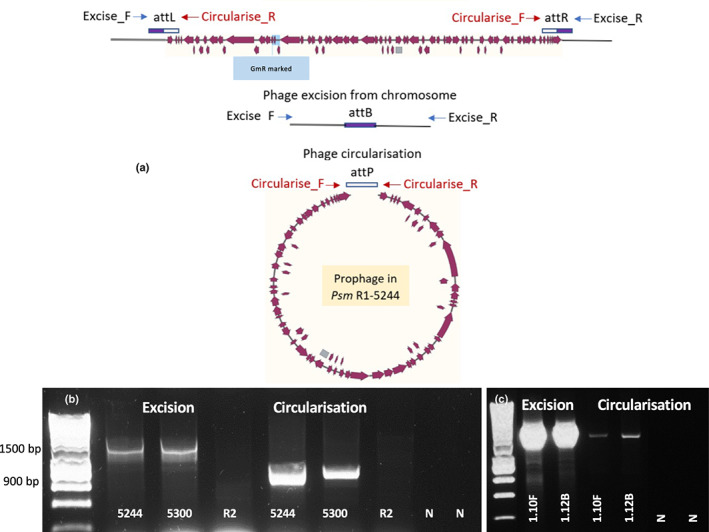
Excision and circularisation of the prophage harbouring *hopAR1* from *Psm* R1 and epiphytic phylogroup four isolates. (a) Schema of prophage excision from the host chromosome and circularisation occurring within bacterial cultures without induction treatments. Phage and bacterial attachment sites (*attP* and *attB*) are marked. Excision was detected using primers Excise F and R located in the bacterial chromosome; no product was expected in the absence of excision. Circularisation was detected using Circularise F and R, amplifying across the *attP* site. (b) Polymerase chain reaction products indicate prophage excision and circularisation occurring from *Psm* R1 strains 5244 and 5300. *Psm* race 2 (R2‐leaf) lacking this *hopAR1* prophage was used as a bacterial control. N, no template control; sizing hyperLadder 1 kb (Bioline, London, UK). (c) Prophage excision from the host chromosome and circularisation in phylogroup 4 strains 1‐10F and 1‐12B.

To examine whether the *Psm* R1 prophage carrying *hopAR1* was intact and mobile, PCRs were performed across the predicted excision site to detect circularisation and excision (Fig. 5a). The prophage genome was found to excise from the chromosome and circularise in *Psm* R1‐5244 and its close relative *Psm* R1‐5300 (a pathogen of plum), which has an almost identical prophage sequence (Fig. 5a,b). The *hopAR1* prophages in the phylogroup 4 isolates (1‐10F and 1‐12B) were also found to excise and circularise, using primers specific to the 1‐12B attachment site locations (Fig. 5c). Sequencing of PCR products confirmed that the bands corresponded to the correct sites overlapping *attL* and *attR*. It was somewhat surprising that 1‐10F, which has a divergent terminal prophage sequence, was able to generate a band for circularisation and excision. Subsequent examination of the prophage region and genome sequence of 1‐10F revealed a region homologous to the 1‐12B prophage *attR* end in another part of the genome (Fig. S3) with identical transposase sequence and *attR*. Whether this represents a real transposition of sequence or a genome misassembly due to the repetitive transposase warrants further investigation. However, the presence of PCR bands for excision and circularisation suggests that the prophage may also be complete in 1‐10F.

### The prophage was induced by various stimuli

**Fig. 6:**
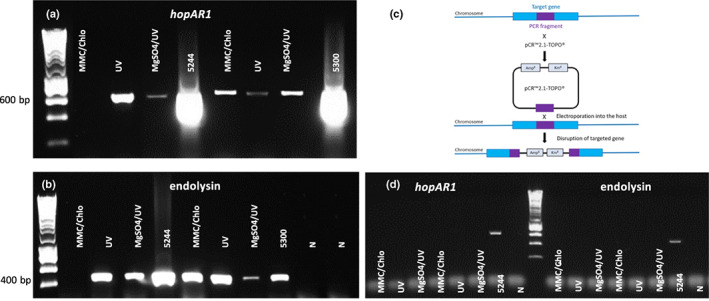
Induction of the *hopAR1*‐encoding prophage in *Psm* R1 is dependent on a functional phage integrase gene. (a, b) Detection of polymerase chain reaction products for *hopAR1*, and the phage endolysin gene from purified phage preparations after induction of *Psm* R1‐5244 and R1‐5300, following mitomycin C (MMC) and Chloroform (Chlo), UV radiation and MgSO_4_ wash and UV radiation treatments. (c) A diagram of the gene knockout strategy. (d) Absence of induction after disruption of the integrase gene in *Psm* R1‐5244 prophage. Polymerase chain reaction analysis was performed for *hopAR1* and phage endolysin genes after induction and purification as in (a). Amplification from bacterial DNA of 5244 and 5300 is shown as positive controls. N, no template control, sizing hyperLadder 1 kb (Bioline).

To examine the activity of the prophage, we tested its inducibility (phage production) by treating bacteria with MMC and chloroform, UV radiation or MgSO_4_ wash plus UV radiation. DNA extractions were performed from the bacterial lysates, and PCR was used to detect *hopAR1* and a phage gene (endolysin). This showed that the prophage was inducible in both *Psm* R1 strains and that the UV treatment was the most effective (Fig. 6a,b). Polymerase chain reaction amplification of a bacterial housekeeping gene (*gyrB*) was also included to rule out any bacterial chromosome contamination of lysate preparations (Fig. S4). To confirm the induction of prophage and further eliminate the possibility of chromosomal contamination leading to false‐positive PCR amplifications, we mutated the second integrase gene next to *attR* in the prophage region of *Psm* R1‐5244 (Fig. 4c) that could be required for the excision process (using a strategy illustrated in Fig. 6c). Prophage induction was then repeated, and PCR was performed for *hopAR1* and the phage endolysin genes; the prophage in *Psm* R1‐5244 did not induce after mutation of the integrase gene (Fig. 6d).

### Prophage transfer between isolates

We next examined whether the prophage transferred between *Psm* R1 and an epiphyte that lacks *hopAR1*. We chose a member of phylogroup 10 (3‐7F), as this clade was frequently found on cherry, but strains do not possess *hopAR1*. The *Psm* R1‐5244 prophage carrying *hopAR1* was marked with a Gentamicin resistance (*Gm* ^*R*^) cassette. To first test whether a recipient strain could integrate the prophage into its chromosome, phage DNA was extracted from induced *Psm* R1‐5244_*Gm* ^*R*^ cells and confirmed to be free of chromosomal DNA. Electroporation (transfection) into competent cells of *P. syringae* 3‐7F_Rifampicin resistant (*Rif*) was carried out using prophage DNA.

Acquisition of the prophage by transfection of 3‐7F_Rif was confirmed by colony PCR using primers for *hopAR1*, the phage endolysin gene and *hopY1* (a T3SE gene present on the chromosome of 3‐7F_*Rif* to confirm its identity). The prophage was subsequently induced from the recipient *P. syringae* 3‐7F*_Rif* electroporant, DNA extracted from lysates and identified by PCR of *hopAR1* (Fig. S5). Thus, the prophage was found to move to *P. syringae* 3‐7F*_Rif* following electroporation.

**Fig. 7:**
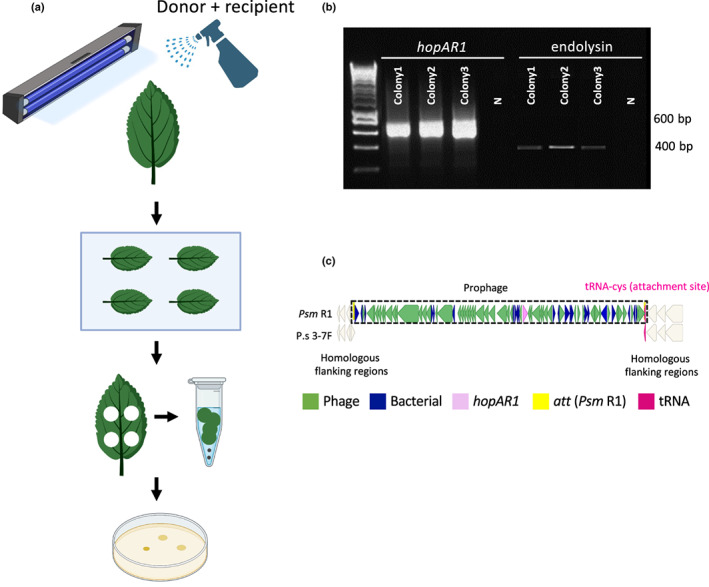
Transfer of the *hopAR1*‐encoding prophage from *Psm* R1‐5244_Gm^*R*^ into *Pseudomonas syringae* phylogroup 10 strain 3‐7F*_Rif* on cherry leaves following UV radiation. (a) Schema of leaf inoculation with *Psm* R1‐5244_*Gm* ^*R*^ (donor) and *Ps* 3‐7F*_Rif* (recipient), irradiation with UV and bacterial isolation. (b) Three Rif‐r Gm‐r resistant colonies were checked by polymerase chain reaction for the *hopAR1* and endolysin genes, which are present in the prophage genome (and for *hopY1*, only present in 3‐7F, Supporting Information Fig. S6B). (c) Alignment of the tRNA‐cys region in *Psm* R1‐5244 and *Ps* 3‐7F to show site of integration into *Ps* 3‐7F. The tRNA‐cys and flanking regions are similar. The DNA alignment is colour‐coded with the key presented (green, phage; blue, bacterial; pink, *hopAR1*; white, flanking genes; yellow, attachment (*att*) sites).

A key test for ecologically relevant HGC is to demonstrate gene exchange *in planta*. To explore gene exchange on the leaf surface, a mixed inoculum of *Psm* R1‐5244_*Gm* ^*R*^ and *P. syringae* 3‐7F*_Rif* was sprayed onto detached cherry leaves (Fig. 7a) and treated with UV radiation or left untreated. GmR colonies of 3‐7F*_Rif* were obtained only in UV‐treated mixed inoculum samples and no other treatments. 3‐7F*_Rif‐Gm* colonies were confirmed to contain the prophage by PCR of *hopAR1*, the endolysin gene (Fig. 7b) and *hopY1* (only in 3‐7F, Fig. S6). To determine where in the *P. syringae* 3‐7F*_Rif* genome the prophage recombined, a homologous tRNA was identified using blastn. Polymerase chain reaction amplifications were then performed using primers for *P. syringae* 3‐7F excision and *Psm* R1‐5244 circularisation region to confirm integration of the phage sequence (Fig. S6). *Pseudomonas syringae* 3‐7F*_Rif* has a homologous tRNA‐cys gene to the prophage attachment site in *Psm* R1‐5244 (Fig. 7c). The prophage integrated into this region in *P. syringae* 3‐7F*_Rif* and was able to excise and circularise (Fig. S6c,d) and be induced (Fig. S6e,f). These results confirmed that environmental conditions can trigger phages to move between strains of *P. syringae* within the phylloplane, actively transferring virulence genes within epiphytic bacterial populations. We have therefore confirmed the bioinformatics‐based predictions of phage activity and HGT.

## Discussion

The sampling conducted in this study presents a snapshot of the *P. syringae* strains occupying the symptomless cherry tree phyllosphere in the UK in 2017. We sampled across regions, orchards, cherry varieties and tissue types with multiple replications to provide a large dataset. Initial phylogenetics and species characterisation by ANI showed clear regional variations in *Pseudomonas* populations, with the location being the major factor determining which members of the *P. syringae* species complex were present. Regional differences may be due to variation in climate, with average temperatures highest in the Southeast. The four cherry varieties sampled are all susceptible to canker pathogens, but Sweetheart and Penny had higher levels of a *Pss* phylogroup 2 strains. There could be subtle differences between varieties that impose selection on particular genotypes in epiphytic bacterial populations. By contrast, host tissue (leaves or woody stem) did not appear to affect the members of the *P. syringae* species complex isolated. Further examination of populations throughout the year may have revealed additional variations and the impact of different environmental and host factors. A recent study on kiwifruit showed that *P. syringae* populations dynamically shift through the growing season (Figueira *et al*., 2020). On a perennial tree crop such as cherry, the resident microflora overwintering on woody tissues may make an important, annually repeated contribution to the initial leaf surface population.

The sequenced epiphytic strains were predominantly from two major groups within the *P. syringae* species complex, phylogroup 10 from the Southeast and phylogroup 2 from the Southwest. Most strains isolated from the West Midlands were from diverse species outside of the known *P. syringae* phylogroups. Such strains do not possess a canonical *hrp* gene‐encoded T3SS (Table S6) and thus are unlikely to cause disease. The frequency of the outlying isolates (representing *c*. 50% of all genomes sequenced) indicates that diverse pseudomonads lacking the T3SS but related to *P. syringae* phylogroups are common and successful epiphytes and share the phyllosphere niche with pathogenic lineages.

Focusing on the major *P. syringae* phylogroups, a large clade of new strains was identified within phylogroup 10 and four strains were from phylogroup 4. Members of these phylogroups have not been isolated from *Prunus* species before. We also isolated new strains from phylogroups 1, 2 and 7, which clustered with known *Prunus* pathogens (*Pss*, *P. s*. pv *avii* and *P. viridiflava*). Intriguingly, the sampling did not find any strains of *Psm* R1 or *Psm* R2, which have been reported as the cherry pathogens most common in the UK (Vicente & Roberts, 2007). A detached leaf pathogenicity test of 14 representative strains indicated that the new members of phylogroup 10 were potentially pathogenic. Further characterisation of this group as pathogens, including whole‐tree inoculations, will be important to determine whether they are active pathogens in the field and not just under optimised laboratory conditions. Our results show that the diversity of pathogens affecting a host plant may be broader than previously realised, a finding that has implications for understanding the emergence of new disease outbreaks.

The diverse surface populations of pseudomonads on cherry occupy a similar niche to pathogenic strains and thus may either be donors or recipients of bacterial genes moving by HGT. Such genes could include those that provide increased fitness on plant hosts such as *Prunus*. Horizontal gene transfer drives bacterial evolution and promotes the rapid emergence of novel pathogenic traits (Gyles & Boerlin, 2014). Strains of *P. syringae* are known to occupy many ecosystems linked to the water cycle, and gene exchange of effectors and other virulence factors is likely to occur in these niches. The plant surface can provide a selective environment in which genes encoding effectors may be adaptive and will therefore increase in frequency in bacterial populations if selected. Only a few steps may be required for an increase in virulence in *P. syringae* populations on plants (Pitman *et al*., 2005; McCann *et al*., 2013; Bartoli *et al*., 2015).

The epiphytic strains from cherry had T3SE and toxin repertoires characteristic of their respective phylogroups. It would appear that a potent mix of T3SEs and toxins may occur on the leaf surface with strains carrying different virulence factors. The isolation of pathogenic *P. syringae* from cankers in *Prunus* spp. has focused on the recovery of single strains of the pathogen and the fulfilment of Koch's postulates by reinoculation (Crosse, 1966; Hulin *et al*., 2018b, 2020). The presence of epiphytic strains harbouring different sets of T3SEs raises the possibility that disease might often be caused by a mixed infection of two or more nonpathogenic strains. Such synergism has been well demonstrated in studies of pear fruit infection by the fireblight pathogen *Erwinia amylovora* (Bennett, 1980). Effector proteins have been considered as ‘public goods’ within mixed populations of bacteria (Smith & Schuster, 2019; Friesen, 2020). The potential for epiphytic strains to act synergistically in mixtures, sharing ‘public goods’, such as T3SEs and toxins, and complementing individual deficiencies in determinants of virulence to cherry merits further exploration.

Our previous work had shown cherry pathogens possess *hopAR1* on prophage sequences, and we have confirmed a similar colocation in epiphytic isolates and other *P. syringae* genomes. A phylogeny based on the HopAR1 protein sequence indicated that the alleles in phylogroup 4 were closely related to those in the phylogroup 3 strain *Psm* R1. This evidence, as well as synteny found in the prophage region, indicates that bacteriophage may have been involved in the transfer of this effector between phylogroups.

The prophage regions harbouring *hopAR1* within *Psm* R1 and one of the phylogroup 4 strains (1‐12B) were predicted to be active, having the required phage machinery and attachment sites. We therefore tested their capacity to transfer genes. The prophages in *P. syringae* excised from the chromosome and circularised in bacterial cells and were induced by treatments with MMC and chloroform or exposure to UV radiation. Similar induction treatments have been used for many other prophages including those in *E. coli* O157 (Asadulghani *et al*., 2009).

Transfer of *hopAR1* was confirmed by tagging the prophage with a Gm resistance marker gene. Transfection was first demonstrated *in vitro* following electroporation of phage DNA. Transfer between strains of *P. syringae* on the leaf surface was confirmed following the treatment of cherry leaves with UV after inoculation with a mixture of phage donor *Psm* R1‐5244_*Gm* ^*R*^ and recipient strain 3‐7F. Our results showed that prophage can actively mediate transfer of genes between bacteria not only *in vitro* but also in their natural environment. Our experiments to demonstrate the transfer of *hopAR1* mirror those described by Asadulghani *et al*. (2009) who reported phage‐mediated transfer occurring even by potentially inactive prophage in *E. coli* O157 *in vitro*.

Our experiments have revealed a remarkable genomic diversity within epiphytic populations of *P. syringae* and confirm that the plant surface niche provides a dynamic environment for gene exchange and the possibility of the emergence of virulent strains capable not only of infecting cherry but also of potentially jumping to other host plants. Further understanding of the relationship between the reservoirs of *P. syringae* on plants and in the wider environment and the emergence of new strains of pathogens should improve rational approaches to disease control. Monitoring the epiphytic microflora for virulence factors would allow prediction of the likelihood of emergence of new pathogens and, through epidemiological surveillance, allow the application of measures for disease control through the modification of agronomic practices. For example, the growth of cherry trees under cover to reduce or eliminate UV‐induction of phage movement might help to limit the spread of phage and virulence genes.

## Competing interests

None declared.

## Author contributions

MTH, MR, JWM, RWJ and RJH conceived the study. MTH and SB performed the sampling and isolation of epiphytes. MTH and AVD carried out the genome sequencing. MTH performed bioinformatics analysis. MR and PS carried out the prophage study. MTH, MR, ZZ, AVD, SB, PS, JWM, RWJ and RJH contributed to the experimental design, interpretation of results and preparation of the article and have approved the final manuscript. MTH and MR contributed equally to this work.

## Supporting information


**Fig. S1** Sampling strategy used in this study.
**Fig. S2** Alignment of *hopAR1*‐containing prophages in *Psm* R1 phylogroup 3 members in comparison with phylogroup 4.
**Fig. S3** The *hopAR1*‐containing prophage region in phylogroup 4 strains 1‐12B and 1‐10F.
**Fig. S4** Induction of the *hopAR1* prophage in *Psm* R1 housekeeping gene check.
**Fig. S5** Transfer of the *hopAR1*‐encoding prophage after electroporation of prophage DNA from *Psm* R1‐5244 into *P*. *syringae* phylogroup 10 strain 3‐7F_*Rif*.
**Fig. S6** Transfer of the *hopAR1*‐encoding prophage from *Psm* R1‐5244_*Gm* ^*R*^ into *P. syringae* phylogroup 10 strain 3‐7F_*Rif* on cherry leaves following UV radiation.
**Table S1** Plasmids used in this study.
**Table S2** Primers used in this study.
**Table S3** Table of randomisation strategy.
**Table S4** Sampling design of this study with no. trees per variety sampled in each orchard.
**Table S5** Genome assemblies generated in this study with statistics and accession information.

**Table S6** Full trait data for strains sequenced in this study and additional genomes.

**Table S7** Results of Prophage Hunter analysis for *hopAR1* gene‐containing regions.
Please note: Wiley is not responsible for the content or functionality of any Supporting Information supplied by the authors. Any queries (other than missing material) should be directed to the *New Phytologist* Central Office.

## Data Availability

The datasets supporting the conclusions of this article are included within the article and its [Link], [Link], [Link].
