# Supplementary material for: Genomic and functional analysis of phage‐mediated horizontal gene transfer in Pseudomonas syringae on the plant surface
Source: New Phytol. 2022 Dec 2;237(3):959–73. doi: 10.1111/nph.18573 (PMC10107160; doi:10.1111/nph.18573)
Supplement: Supplementary file 1 — Fig. S1 Sampling strategy used in this study. Fig. S2 Alignment of hopAR1‐containing prophages in Psm R1 phylogroup 3 members in comparison with phylogroup 4. Fig. S3 The hopAR1‐containing prophage region in phylogroup 4 strains 1‐12B and 1‐10F. Fig. S4 Induction of the hopAR1 prophage in Psm R1 housekeeping gene check. Fig. S5 Transfer of the hopAR1‐encoding prophage after electroporation of prophage DNA from Psm R1‐5244 into P. syringae phylogroup 10 strain 3‐7F_Rif. Fig. S6 Transfer of the hopAR1‐encoding prophage from Psm R1‐5244_Gm R into P. syringae phylogroup 10 strain 3‐7F_Rif on cherry leaves following UV radiation. Table S1 Plasmids used in this study. Table S2 Primers used in this study. Table S3 Table of randomisation strategy. Table S4 Sampling design of this study with no. trees per variety sampled in each orchard. Table S5 Genome assemblies generated in this study with statistics and accession information. [file NPH-237-959-s003.pdf]

New Phytologist Supporting Information

Article title:

Genomic and functional analysis of phage mediated horizontal gene transfer in *Pseudomonas syringae* on the plant surface

Authors:

Michelle T. Hulin[1†#], Mojgan Rabiey[2†], Ziyue Zeng[1], Andrea Vadillo Dieguez[1], Sophia Bellamy[1], Phoebe Swift[2], John W. Mansfield[3], Robert W. Jackson[2*] and Richard J. Harrison[1*‡]

[1]NIAB, Lawrence Weaver Road, Cambridge, CB3 0LE, UK

[2]School of Biosciences and the Birmingham Institute of Forest Research, University of Birmingham, Birmingham, B15 2TT, UK

[3]Faculty of Natural Sciences, Imperial College London, London, SW7 2BX, UK

[†]These first authors contributed equally to this work.

# Present address: The Sainsbury Laboratory, Norwich, NR4 7UH, UK

[‡] Present address: Plant Science Group, Wageningen University and Research, Wageningen, 6708WB, The Netherlands

*Joint corresponding authors: Robert W Jackson (r.w.jackson@bham.ac.uk) and Richard J. Harrison (Richard.Harrison@wur.nl)

Article acceptance date: 19th October 2022

Supplementary information includes:

7 Supplementary Tables (5 in PDF, 2 as excel)

6 Supplementary Figures

Supplementary Tables

Table S1. Plasmids used in this study

Plasmids	Host	Detail	Reference
pRK2073	*E. coli*	Helper plasmid, Spc^r, $Strep^r$	Leong *et al.*, 1982
pTS-1	*E. coli*	pME3087 derivative containing a *sacB* counter-selection marker	Scott *et al.*, 2017

Table S2. Primers used in this study

Primer name	Sequence	Reference	Size (bp)	Tm (°C)
Excise_prophage_F	ATGTCTACGACGATGACCGC	This study	1493	55
Excise_prophage_R	GAAAACGTCGGTAATCGCGG			
Circularise_prophage_F	ACGTTATCGCGATGAACCGA	This study	904	55
Circularise_prophage_R	TCGGATGATCTCAACGCGAG			
Excise_3.7F_F	ATGTCGACGACGATGACGGC	This study	1325	58
Excise_3.7F_R	GATGACGTCGCCAACCGAGG			
Excise_1.12B_F	CGTTCTGGTCAACAATGCCG	This study	1097	58
Excise_1.12B_R	AGGGTAATGTCGTCACGCAG			
Circularise_1.12B_R	AAGGTCGTTGGTGAAGCGAT	This study	1172	58
Circularise_1.12B_F	ACTTTCAAGCCGTTGGGTGA			
hopAR1_gene_F	CGAGCCGAGAAATGCCTTTA	This study	632	55
hopAR1_gene_R	AAGCATCTGAGGTGCTGCAC			
*hopY1*_F	CAGTATCGAGAAAGGGAAACTGTTC	This study	153	60
*hopY1*_R	ATATAAGCCCGGACAAGGTTCAAG			
prophage_endolysin_F	ATGGTCAGAAAGTCGCCCTT	This study	425	55
prophage_endolysin_R	CTGCAGATCCTCCCGAACG			
gyrB _F	CAGGAAACAGCTATGACCAYGSNGGNGGNAARTTYRA	Yamamoto et al. 2000	900	60
*gyrB*_R	TGTAAAACGACGGCCAGTGCNGGRTCYTTYTCYTGRCA			
Integrase-middle_F	TCCCGTCTAACCATTCGTGC	This study	384	55
Integrase-middle_R	TCATTCCTGCCGAGAACACC			
M13_F	GTAAAACGACGGCCAG	This study	400	55
M13_R	CAGGAAACAGCTATGAC			
Prophage_*Gm*_F	AAGCTTCATGTGCCTGGAGA	This study	1817	55

Prophage_*Gm*_R	GCGCCTCCAGAGCAAACCAC			
Gm-cassette_F	CAGCAACGACGTAACACAGC	This study	244	58
Gm-cassette_R	TTCTTGGTCGAAGGCAGCAA			
Ps-genus-F	GACGGGTGAGTAATGCCTA	Spilker et al. 2004	618	54
Ps-genus-R	CACTGGTGTTCCTTCCTATA			

Table S3. Table of randomisation strategy. A random subset of two samples each from four trees per variety per orchard was selected for the subset. The final 166 strains genome sequenced were after *Pseudomonas* identification. SH: Sweetheart, Ko: Kordia, La: Lapins, Pe: Penny

**Orchard**	**Location**	**No. trees SH**	**No. trees Ko**	**No. trees La**	**No. trees Pe**	**Total Trees**	**Total isolates**	**Present**	**% Present**	**Subset trees**	**Subset samples**	**Final subset**	**% Subset**
H	Southeast	7	7	7	7	28	336	164	48.81	16	32	28	87.5
M	Southeast	7	7	7	7	28	336	123	36.61	16	32	13	40.6
A	Southeast	7	7	7	7	28	336	227	67.56	16	32	31	96.9
Lo	West Midlands	7	7	7	7	28	336	151	44.94	16	32	4	12.5
L	West Midlands	7	7	7	7	28	336	100	29.76	16	32	16	50
Ha	West Midlands	7	7	7	NA	21	252	94	37.30	12	24	12	50
N	South West	7	7	NA	7	21	252	159	63.10	12	24	22	91.7
W	South West	7	7	7	2	23	276[a]	245	88.77	14	28	23	82.1
B	South West	7	NA	7	7	21	252	239	94.84	12	24	17	70
						226	2712	1502	55.38	130	260	**166**	63.8

[a] There were four cultivars but one only had two trees resulting in fewer overall samples

Table S4. Sampling design of this study with number of trees per variety sampled in each orchard

Orchard	Location	SWEETHEART	KORDIA	LAPINS	PENNY
1	Southeast	7	7	7	7
2	Southeast	7	7	7	7
3	Southeast	7	7	7	7
4	West Midlands	7	7	7	7
5	West Midlands	7	7	7	7
6	West Midlands	7	7	7	NA
7	South West	7	7	NA	7
8	South West	7	7	7	2
9	South West	7	NA	7	7

Table S5: Genome assemblies generated in this study with statistics and accession information. N50: sequence length of the shortest contig at 50% of assembly length. These are all listed under BioProject PRJNA587608

Strain	Species	Biosample	Sequence coverage	Contigs	Total length	GC (%)	N50	Accession
PA-1-10F	*Pseudomonas tremae*	SAMN13195884	22.27	161	5812802	57.72	112535	WJYQ00000000
PA-1-10F (minION)	*Pseudomonas tremae*	SAMN17034053	172	3	5,887,743	57.50	5717648	CP066270-2
PA-1-11B	*Pseudomonas syringae*	SAMN13195885	19.91	92	5984305	59.06	123235	WJYR00000000
PA-1-11C	*Pseudomonas syringae*	SAMN13195886	18.14	177	5977542	59.07	75089	WJYS00000000
PA-1-11E	*Pseudomonas syringae*	SAMN13195887	20.09	108	5978109	59.07	115302	WJYT00000000
PA-1-11G	*Pseudomonas syringae*	SAMN13195888	19.6	99	5983784	59.07	106533	WJYU00000000
PA-1-12B	*Pseudomonas tremae*	SAMN13195889	17.93	306	5870653	57.67	41013	WJYV00000000
PA-1-12B (minION)	*Pseudomonas tremae*	SAMN17034054	53	3	5,956,622	57.50	5876612	CP066267-9
PA-1-12H	*Pseudomonas syringae*	SAMN13195890	20.42	171	6034432	58.83	83510	WJYW00000000
PA-1-1C	*Pseudomonas tremae*	SAMN13195891	27.13	186	5839724	57.78	71502	WJYX00000000
PA-1-1E	*Pseudomonas syringae*	SAMN13195892	22.13	66	5973823	59.33	181246	WJYY00000000
PA-1-1G	*Pseudomonas tremae*	SAMN13195893	18.38	156	5844547	57.76	120777	WJYZ00000000
PA-1-2A	*Pseudomonas sp.*	SAMN13195894	15.92	376	6019179	60.11	31933	WJZA00000000
PA-1-3B	*Pseudomonas syringae*	SAMN13195895	20.65	213	6550483	59.15	67862	WJZB00000000
PA-1-3C	*Pseudomonas syringae*	SAMN13195896	9.54	822	5963731	60.15	11275	WJZC00000000

PA-1-3F	*Pseudomonas sp.*	SAMN13195897	23.07	89	5944016	60.14	145109	WJZD00000000
PA-1-3G	*Pseudomonas syringae*	SAMN13195898	12.84	275	5554605	58.93	40767	WJZE00000000
PA-1-4C	*Pseudomonas syringae*	SAMN13195899	23.05	79	5560365	58.93	158777	WJZF00000000
PA-1-4E	*Pseudomonas syringae*	SAMN13195900	10.26	849	5956064	60.1	11857	WJZG00000000
PA-1-4G	*Pseudomonas syringae*	SAMN13195901	24.41	53	5562794	58.93	203179	WJZH00000000
PA-1-4H	*Pseudomonas syringae*	SAMN13195902	10.5	995	5489372	58.92	9501	WJZI00000000
PA-1-5A	*Pseudomonas sp.*	SAMN13195903	14.35	501	6016809	60.11	19601	WJZJ00000000
PA-1-5B	*Pseudomonas syringae*	SAMN13195904	13.88	578	5990301	60.11	16348	WJZK00000000
PA-1-6A	*Pseudomonas sp.*	SAMN13195905	20.51	185	6030415	60.11	65397	WJZL00000000
PA-1-6B	*Pseudomonas sp.*	SAMN13195906	30.7	137	6030995	60.11	85355	WJZM00000000
PA-1-6G	*Pseudomonas sp.*	SAMN13195907	18.77	225	6029244	60.11	51556	WJZN00000000
PA-1-7A	*Pseudomonas syringae*	SAMN13195908	13.55	505	6014741	60.11	20205	WJZO00000000
PA-1-7C	*Pseudomonas syringae*	SAMN13195909	15.26	400	6311622	59.46	35932	WJZP00000000
PA-1-7F	*Pseudomonas syringae*	SAMN13195910	15.22	425	6019096	60.11	27133	WJZQ00000000
PA-1-8B	*Pseudomonas poae*	SAMN13195911	12.96	312	5544495	60.88	31916	WJZR00000000
PA-1-8C	*Pseudomonas sp.*	SAMN13195912	20.54	165	6030665	60.11	72191	WJZS00000000
PA-2-10C	*Pseudomonas syringae*	SAMN13195913	12.28	147	5560226	58.93	66866	WJZT00000000
PA-2-10E	*Pseudomonas syringae*	SAMN13195914	15.6	129	5561355	58.93	73612	WJZU00000000
PA-2-11B	*Pseudomonas syringae*	SAMN13195915	17.36	117	5561938	58.93	97916	WJZV00000000
PA-2-11D	*Pseudomonas syringae*	SAMN13195916	19.65	98	5569021	58.93	120482	WJZW00000000
PA-2-1F	*Pseudomonas poae*	SAMN13195917	14.85	298	5558521	60.94	40935	WJZX00000000
PA-2-2B	*Pseudomonas marginalis*	SAMN13195918	18.78	433	6596420	60.38	27000	WJZY00000000
PA-2-2G	*Pseudomonas syringae*	SAMN13195919	11.13	682	5481432	59.01	16068	WJZZ00000000
PA-2-3C	*Pseudomonas syringae*	SAMN13195920	18.58	231	6223737	58.99	62557	WKAA00000000
PA-2-4G	*Pseudomonas syringae*	SAMN13195921	18.76	288	5568002	58.95	60662	WKAB00000000
PA-2-4H	*Pseudomonas syringae*	SAMN13195922	12.02	298	5553622	58.93	36815	WKAC00000000
PA-2-5A	*Pseudomonas syringae*	SAMN13195923	11.93	265	5559251	58.92	37570	WKAD00000000
PA-2-5E	*Pseudomonas syringae*	SAMN13195924	7.22	685	5502601	58.98	12858	WKAE00000000
PA-2-6C	*Pseudomonas syringae*	SAMN13195925	22.23	128	5559310	58.93	89509	WKAF00000000
PA-2-6D	*Pseudomonas syringae*	SAMN13195926	20.36	146	5560720	58.93	68237	WKAG00000000
PA-2-7C	*Pseudomonas syringae*	SAMN13195927	18.54	159	5563741	58.93	70217	WKAH00000000
PA-2-7E	*Pseudomonas syringae*	SAMN13195928	19.55	87	5562874	58.92	141234	WKAI00000000
PA-2-7G	*Pseudomonas syringae*	SAMN13195929	12.01	472	5548110	58.93	21596	WKAJ00000000
PA-2-8C	*Pseudomonas syringae*	SAMN13195930	22.85	171	5550618	58.96	73153	WKAK00000000
PA-2-9E	*Pseudomonas syringae*	SAMN13195931	14.16	192	5558569	58.92	53678	WKAL00000000
PA-2-9G	*Pseudomonas syringae*	SAMN13195932	13.37	277	5562874	58.94	39698	WKAM00000000
PA-3-10B	*Pseudomonas syringae*	SAMN13195933	20.68	75	5563512	58.93	177401	WKAN00000000
PA-3-10C	*Pseudomonas sp.*	SAMN13195934	16.43	611	6767337	59.35	22685	WKAO00000000
PA-3-10H	*Pseudomonas syringae*	SAMN13195935	15.65	135	5569082	58.93	97832	WKAP00000000
PA-3-11B	*Pseudomonas syringae*	SAMN13195936	11.77	613	5515966	58.98	15231	WKAQ00000000
PA-3-11C	*Pseudomonas sp.*	SAMN13195937	15.19	716	6699964	59.43	17386	WKAR00000000
PA-3-1G	*Pseudomonas syringae*	SAMN13195938	14.79	143	5561117	58.93	70881	WKAS00000000
PA-3-2A	*Pseudomonas salomonii*	SAMN13195939	18.45	192	6472686	60.19	71976	WKAT00000000

PA-3-2D	*Pseudomonas syringae*	SAMN13195940	18.16	92	5562802	58.92	118289	WKAU00000000
PA-3-2F	*Pseudomonas syringae*	SAMN13195941	20.6	62	5561061	58.93	177401	WKAV00000000
PA-3-3C	*Pseudomonas syringae*	SAMN13195942	15.46	232	6091761	59.21	45104	WKAW00000000
PA-3-3G	*Pseudomonas syringae*	SAMN13195943	18.44	126	5567882	58.93	86091	WKAX00000000
PA-3-4D	*Pseudomonas syringae*	SAMN13195944	15.14	152	5563296	58.93	74211	WKAY00000000
PA-3-4E	*Pseudomonas syringae*	SAMN13195945	26	89	5570531	58.93	176576	WKAZ00000000
PA-3-5D	*Pseudomonas sp.*	SAMN13195946	34.34	263	6766636	59.25	66335	WKBA00000000
PA-3-5G	*Pseudomonas syringae*	SAMN13195947	10.38	738	5502486	59	12040	WKBB00000000
PA-3-6E	*Pseudomonas sp.*	SAMN13195948	23.45	425	6647149	59.58	39672	WKBC00000000
PA-3-6H	*Pseudomonas sp.*	SAMN13195949	16.34	644	6700806	59.28	21484	WKBD00000000
PA-3-7A	*Pseudomonas syringae*	SAMN13195950	13.87	376	5555591	58.92	25605	WKBE00000000
PA-3-7D	*Pseudomonas syringae*	SAMN13195951	23.09	107	5532845	59.02	148481	WKBF00000000
PA-3-7F	*Pseudomonas syringae*	SAMN13195952	15.45	248	5542655	59	46653	WKBG00000000
PA-3-8H	*Pseudomonas syringae*	SAMN13195953	31.18	51	5563834	58.93	223780	WKBH00000000
PA-3-9A	*Pseudomonas syringae*	SAMN13195954	44.05	47	5565743	58.93	211683	WKBI00000000
PA-3-9F	*Pseudomonas syringae*	SAMN13195955	16.09	113	5562069	58.93	103648	WKBJ00000000
PA-4-10A	*Pseudomonas syringae*	SAMN13195956	17.29	129	5932683	59.31	87333	WKBK00000000
PA-4-10C	*Pseudomonas syringae*	SAMN13195957	15.95	397	6188270	59.08	27227	WKBL00000000
PA-4-10F	*Pseudomonas syringae*	SAMN13195958	23.18	97	5934683	59.3	114209	WKBM00000000
PA-4-11A	*Pseudomonas syringae*	SAMN13195959	12.52	934	5847016	59.47	10509	WKBN00000000
PA-4-12E	*Pseudomonas syringae*	SAMN13195960	18.64	228	6205517	59.07	61185	WKBO00000000
PA-4-12H	*Pseudomonas syringae*	SAMN13195961	17.85	309	5920679	59.41	42774	WKBP00000000
PA-4-2D	*Pseudomonas syringae*	SAMN13195962	18.28	149	5909083	59.34	68138	WKBQ00000000
PA-4-2F	*Pseudomonas syringae*	SAMN13195963	31.71	165	5925842	59.34	71269	WKBR00000000
PA-4-2H	*Pseudomonas syringae*	SAMN13195964	19.99	178	6204792	59.01	68865	WKBS00000000
PA-4-3D	*Pseudomonas syringae*	SAMN13195965	27.5	155	5921483	59.35	82513	WKBT00000000
PA-4-3H	*Pseudomonas syringae*	SAMN13195966	20.54	100	5935660	59.31	113568	WKBU00000000
PA-4-4B	*Pseudomonas syringae*	SAMN13195967	22.25	122	5972595	59.26	96435	WKBV00000000
PA-4-4G	*Pseudomonas syringae*	SAMN13195968	18.27	153	5953074	59.38	116111	WKBW00000000
PA-4-5C	*Pseudomonas syringae*	SAMN13195969	13.84	508	5928644	59.3	20800	WKBX00000000
PA-4-6C	*Pseudomonas syringae*	SAMN13195970	18.72	426	6292395	59.03	67705	WKBY00000000
PA-4-7B	*Pseudomonas syringae*	SAMN13195971	16.05	320	6211872	59.06	36963	WKBZ00000000
PA-4-7C	*Pseudomonas syringae*	SAMN13195972	11.67	391	5921863	59.36	25912	WKCA00000000
PA-4-7F	*Pseudomonas syringae*	SAMN13195973	22.81	164	5919422	61.04	60206	WKCB00000000
PA-4-8B	*Pseudomonas syringae*	SAMN13195974	20.19	167	5931797	59.31	77527	WKCC00000000
PA-4-8C	*Pseudomonas sp.*	SAMN13195975	14.92	604	6522679	60.15	19423	WKCD00000000
PA-4-8H	*Pseudomonas syringae*	SAMN13195976	10.91	741	6154788	59.02	14446	WKCE00000000
PA-4-9B	*Pseudomonas syringae*	SAMN13195977	9.84	685	5887173	59.34	13915	WKCF00000000
PA-5-10A	*Pseudomonas syringae*	SAMN13195978	18.18	153	5934624	59.37	80347	WKCG00000000
PA-5-10B	*Pseudomonas simiae*	SAMN13195979	21.39	144	6219661	60.2	119127	WKCH00000000
PA-5-10D	*Pseudomonas syringae*	SAMN13195980	16.78	174	5934531	59.36	67183	WKCI00000000
PA-5-10E	*Pseudomonas simiae*	SAMN13195981	27.36	84	6230800	60.17	254940	WKCJ00000000
PA-5-10F	*Pseudomonas syringae*	SAMN13195982	26.92	98	5943095	59.31	136184	WKCK00000000

PA-5-10H	*Pseudomonas simiae*	SAMN13195983	27.97	100	6226496	60.17	158826	WKCL00000000
PA-5-11C	*Pseudomonas simiae*	SAMN13195984	19.5	130	6228012	60.16	88322	WKCM00000000
PA-5-11F	*Pseudomonas salomonii*	SAMN13195985	14.59	664	6395144	60.09	22893	WKCN00000000
PA-5-12B	*Pseudomonas lurida*	SAMN13195986	20.66	329	6006098	61.01	68476	WKCO00000000
PA-5-12C	*Pseudomonas lurida*	SAMN13195987	27.42	108	6055691	60.94	166574	WKCP00000000
PA-5-12D	*Pseudomonas simiae*	SAMN13195988	19.49	111	6230751	60.15	105630	WKCQ00000000
PA-5-1G	*Pseudomonas syringae*	SAMN13195989	10.65	655	5889205	59.34	14554	WKCR00000000
PA-5-2A	*Pseudomonas syringae*	SAMN13195990	19.41	105	5938454	59.36	99401	WKCS00000000
PA-5-2C	*Pseudomonas syringae*	SAMN13195991	11.3	418	5921732	59.35	24118	WKCT00000000
PA-5-2F	*Pseudomonas syringae*	SAMN13195992	10.9	527	5917820	59.35	19091	WKCU00000000
PA-5-2G	*Pseudomonas syringae*	SAMN13195993	23.55	163	6071578	59.22	112613	WKCV00000000
PA-5-3A	*Pseudomonas syringae*	SAMN13195994	8.35	751	5874013	59.35	12700	WKCW00000000
PA-5-3C	*Pseudomonas syringae*	SAMN13195995	16.23	321	6023598	59.25	31189	WKCX00000000
PA-5-3F	*Pseudomonas syringae*	SAMN13195996	24.24	104	6058004	59.21	125823	WKCY00000000
PA-5-3G	*Pseudomonas syringae*	SAMN13195997	23.49	99	5965410	59.27	112180	WKCZ00000000
PA-5-4A	*Pseudomonas sp.*	SAMN13195998	23.46	211	6661995	60.01	60000	WKDA00000000
PA-5-4B	*Pseudomonas sp.*	SAMN13195999	22.66	149	6660102	60	79804	WKDB00000000
PA-5-4F	*Pseudomonas sp.*	SAMN13196000	19.9	268	6660883	60.01	40835	WKDC00000000
PA-5-4G	*Pseudomonas sp.*	SAMN13196001	14.96	474	6640952	60.01	24215	WKDD00000000
PA-5-4H	*Pseudomonas sp.*	SAMN13196002	25.25	161	6664747	60.01	73092	WKDE00000000
PA-5-6A	*Pseudomonas syringae*	SAMN13196003	9.8	911	6152059	58.69	12084	WKDF00000000
PA-5-6C	*Pseudomonas syringae*	SAMN13196004	19.78	209	6264062	58.71	97341	WKDG00000000
PA-5-7A	*Pseudomonas syringae*	SAMN13196005	15.68	202	6027823	59.24	56207	WKDH00000000
PA-5-7C	*Pseudomonas syringae*	SAMN13196006	17.98	156	6033970	59.24	72285	WKDI00000000
PA-5-7F	*Pseudomonas syringae*	SAMN13196007	28.35	86	5941005	59.36	154499	WKDJ00000000
PA-5-7H	*Pseudomonas syringae*	SAMN13196008	14.98	259	6023653	59.25	42482	WKDK00000000
PA-5-8G	*Pseudomonas syringae*	SAMN13196009	17.14	166	6032992	59.24	65652	WKDL00000000
PA-5-9C	*Pseudomonas simiae*	SAMN13196010	20.6	103	6227738	60.16	128988	WKDM00000000
PA-5-9D	*Pseudomonas syringae*	SAMN13196011	19.59	127	5935282	59.3	85928	WKDN00000000
PA-6-1D	*Pseudomonas sp.*	SAMN13196012	15.32	356	6531176	59.92	32453	WKDO00000000
PA-6-1H	*Pseudomonas sp.*	SAMN13196013	23.64	156	6538877	59.91	83719	WKDP00000000
PA-6-2A	*Pseudomonas canadensis*	SAMN13196014	15.5	358	6506696	60.17	34971	WKDQ00000000
PA-6-2E	*Pseudomonas sp.*	SAMN13196015	28.73	109	6539456	59.91	121963	WKDR00000000
PA-6-2G	*Pseudomonas congelans*	SAMN13196016	23.7	97	5856292	58.87	199020	WKDS00000000
PA-6-3A	*Pseudomonas syringae*	SAMN13196017	20.15	147	6168668	59.17	102956	WKDT00000000
PA-6-3B	*Pseudomonas lactis*	SAMN13196018	35.62	129	6584463	60.14	148285	WKDU00000000
PA-6-3C	*Pseudomonas sp.*	SAMN13196019	29.34	105	6541362	59.9	112150	WKDV00000000
PA-6-3F	*Pseudomonas sp.*	SAMN13196020	19.5	205	6541048	59.91	71803	WKDW00000000
PA-6-3H	*Pseudomonas syringae*	SAMN13196021	22.39	182	5921741	61.04	57579	WKDX00000000
PA-6-4A	*Pseudomonas lactis*	SAMN13196022	28.77	155	6486246	60.11	113657	WKDY00000000
PA-6-4B	*Pseudomonas lactis*	SAMN13196023	13.67	556	6562822	60.14	20222	WKDZ00000000
PA-6-4C	*Pseudomonas lactis*	SAMN13196024	22	198	6582283	60.14	73743	WKEA00000000
PA-6-4D	*Pseudomonas proteolytica*	SAMN13196025	17.18	529	6620059	60.23	21887	WKEB00000000

PA-6-4F	*Pseudomonas sp.*	SAMN13196026	19.45	220	6417691	60.02	55735	WKEC00000000
PA-6-5B	*Pseudomonas gessardii*	SAMN13196027	47.96	190	6621829	60.52	73368	WKED00000000
PA-6-5F	*Pseudomonas gessardii*	SAMN13196029	17.87	582	6535129	60.7	23236	WKEF00000000
PA-6-6A	*Pseudomonas gessardii*	SAMN13196030	17.93	550	6348205	60.8	37727	WKEG00000000
PA-6-6D	*Pseudomonas gessardii*	SAMN13196031	16.66	903	6514949	60.72	12664	WKEH00000000
PA-6-6F	*Pseudomonas gessardii*	SAMN13196032	30.22	370	6422995	60.85	54712	WKEI00000000
PA-6-6G	*Pseudomonas lurida*	SAMN13196033	33.53	54	6037694	60.94	227950	WKEJ00000000
PA-6-6H	*Pseudomonas syringae*	SAMN13196034	11.31	447	5892726	59.38	23833	WKEK00000000
PA-6-7A	*Pseudomonas lactis*	SAMN13196035	21.44	188	6582447	60.14	75669	WKEL00000000
PA-6-7D	*Pseudomonas lactis*	SAMN13196036	14.05	531	6553902	60.14	29365	WKEM00000000
PA-6-7E	*Pseudomonas lactis*	SAMN13196037	16.48	292	6575743	60.14	44058	WKEN00000000
PA-6-7G	*Pseudomonas lactis*	SAMN13196038	11.5	857	6420292	60.09	11814	WKEO00000000
PA-6-8B	*Pseudomonas lactis*	SAMN13196039	30.01	123	6584105	60.14	114675	WKEP00000000
PA-6-8C	*Pseudomonas syringae*	SAMN13196040	18.76	101	5671921	59.11	118138	WKEQ00000000
PA-6-8E	*Pseudomonas syringae*	SAMN13196041	14.07	214	5670328	59.12	48377	WKER00000000
PA-6-8F	*Pseudomonas lactis*	SAMN13196042	15.64	337	6574960	60.15	36400	WKES00000000
PA-6-8G	*Pseudomonas syringae*	SAMN13196043	14.11	356	5905929	61.04	28091	WKET00000000
PA-6-9A	*Pseudomonas syringae*	SAMN13196044	14.67	296	5910870	61.04	37681	WKEU00000000
PA-6-9D	*Pseudomonas syringae*	SAMN13196045	12.57	448	5899340	61.04	21864	WKEV00000000
PA-6-9F	*Pseudomonas proteolytica*	SAMN13196046	18.82	320	6633661	60.24	39121	WKEW00000000
PA-6-9G	*Pseudomonas lactis*	SAMN13196047	17.15	282	6583499	60.14	39796	WKEX00000000
PA-7-1B	*Pseudomonas simiae*	SAMN13196048	30.45	198	6026817	60.62	102031	WKEY00000000
PA-7-1C	*Pseudomonas syringae*	SAMN13196049	11.87	423	6083695	59.21	27917	WKEZ00000000
PA-7-1E	*Pseudomonas sp.*	SAMN13196050	23.05	142	6420947	60	98859	WKFA00000000

Table S6. Full trait data for strains sequenced in this study and additional genomes, including isolation information, species Average Nucleotide Identity (ANI) groups, type 3 secreted effectors (T3SEs) and toxin BLAST results (see excel)

Table S7. Results of Prophage Hunter analysis for *hopAR1* gene-containing regions (see excel)

Supplementary Figures

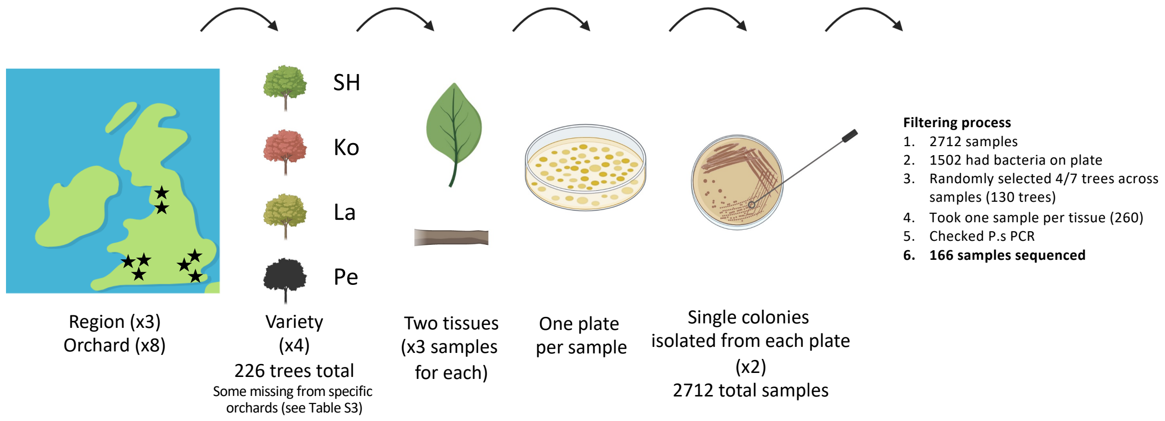

Figure S1: Sampling strategy used in this study to isolate pseudomonads from cherry phyllosphere across the UK and filtering process used to obtain 166 *P. syringae* (*P.s*) strains for genome sequencing that represent the entire sample. Orchards are labelled with stars on a map of the British Isles. Tree varieties are listed: SH: Sweetheart, Ko: Kordia, La: Lapins, Pe: Penny.

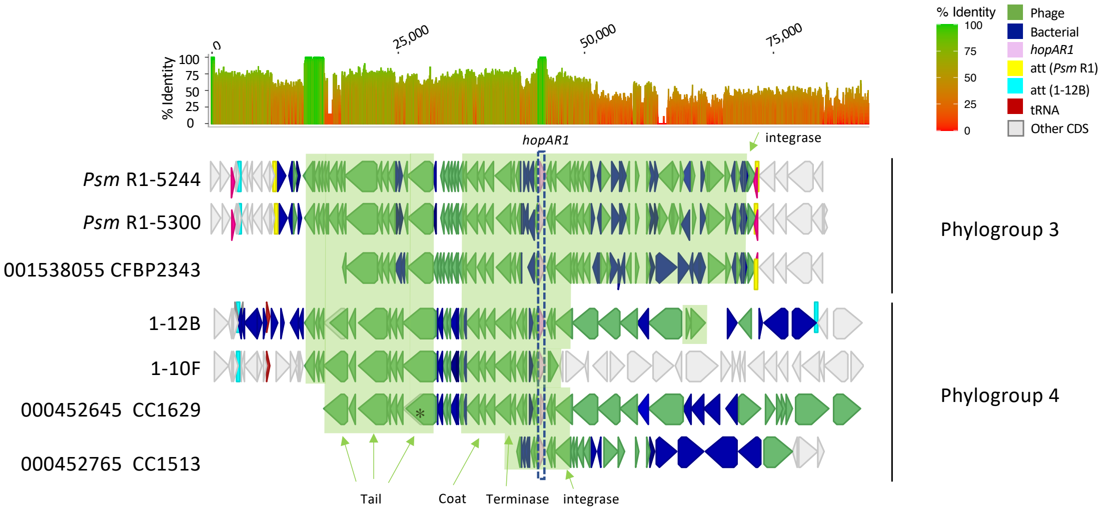

Figure S2. Alignment of *hopAR1*-containing prophages in *Psm* R1 phylogroup 3 members in comparison to phylogroup 4. Syntenic regions are highlighted in green. The key denotes whether genes were annotated as phage or bacterial by PHASTER and other key regions including the predicted attachment (*att*) sites. Similarity between the two sequences is shown on the above plot of identity over sliding windows of 20 nucleotides. The plot is coloured by a scale from red (low) to green (high) identity ranging from 0-100%. Key phage genes and *hopAR1* are highlighted in green boxes and a dashed box respectively.

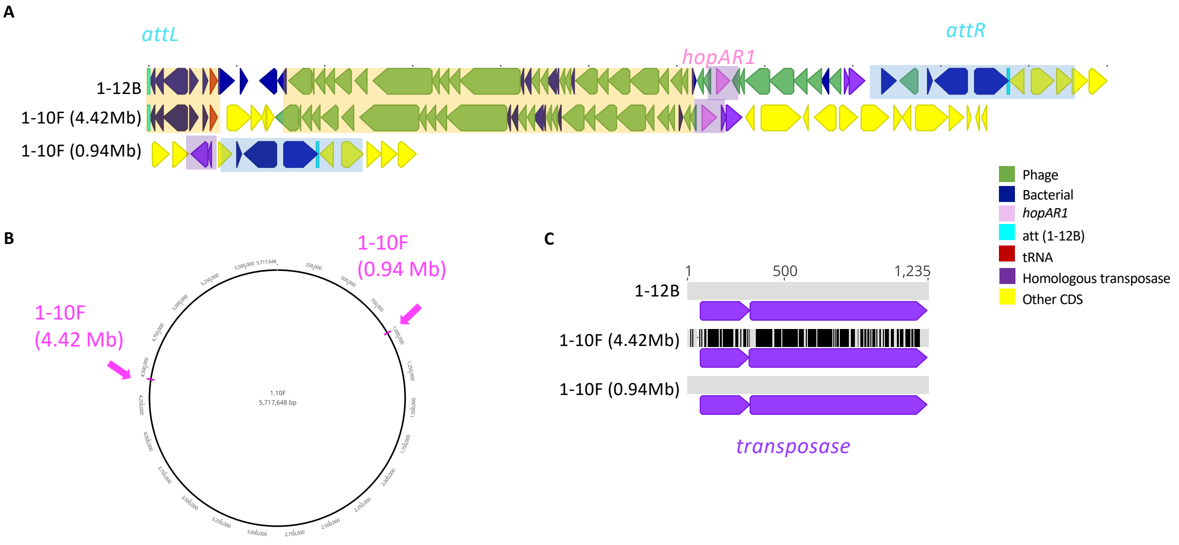

Figure S3. The *hopAR1*-containing prophage region in phylogroup 4 strains 1-12B and 1-10F. Syntenic regions are highlighted in coloured boxes. The key denotes whether genes were annotated as phage or bacterial by PHASTER and other key regions including the predicted attachment sites (*att*). Yellow genes are other CDS not predicted to be part of prophage region. A: Region of 1-12B prophage aligned to 1-10F and an alternative end region from another part of the genome. Location on the 1-10F chromosome is labelled as 4.42Mb or 0.94 Mb. The first 1-10F region at position 4.42 Mb aligns to the first part of the prophage whilst the alternative region at 0.94 Mb aligns to the end of the 1-12B prophage and has the *attR* sequence. B: These locations are shown on a circular diagram of the 1.10F chromosome with arrows. C: Alignment of the homologous transposases show that the transposase in 1-12B and the 1-10F end region are identical supporting the possibility that these regions could be part of a complete 1-10F prophage.

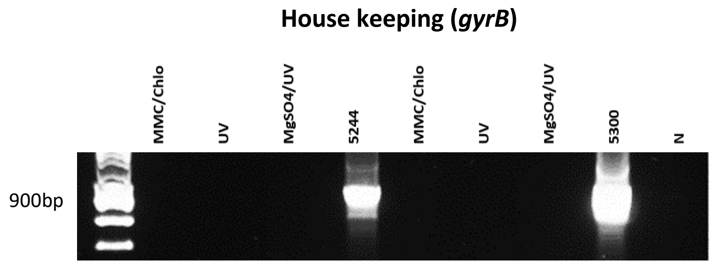

Figure S4. Induction of the *hopAR1* prophage in *Psm* R1 house-keeping gene check. PCR products for the bacterial housekeeping gene (*gyrB*) in purified phage preparations after induction of *Psm* R1-5244 and R1-5300, following mitomycin C (MMC) and Chloroform (Chlo), UV radiation and $MgSO_4$ wash and UV radiation treatments. Failure to amplify *gyrB* confirmed the absence of bacterial chromosomal contamination in the induced prophages. N: no template control, sizing hyperladder 1kb (Bioline, UK).

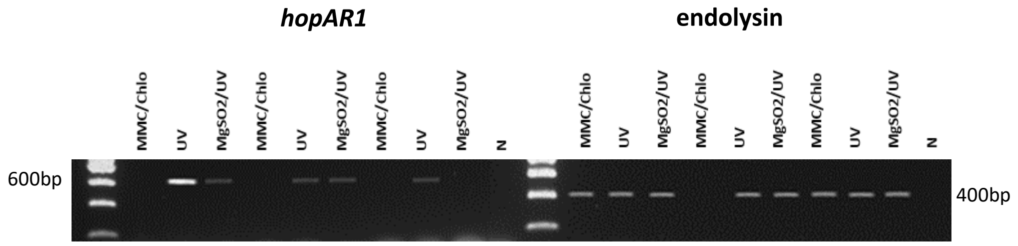

Figure S5. Transfer of the *hopAR1*-encoding prophage after electroporation of prophage DNA from *Psm* R1-5244 into *P. syringae* phylogroup 10 strain 3-7F_*Rif*. PCR products for *hopAR1* and endolysin genes detected in purified phage preparations after induction, following mitomycin C (MMC) and chloroform (Chlo), UV radiation, and $MgSO_4$ wash and UV radiation treatments. Results are from three independent colonies (lanes 1-3, 4-6, 7-9). N: no template control, hyperladder 1kb (Bioline, UK).

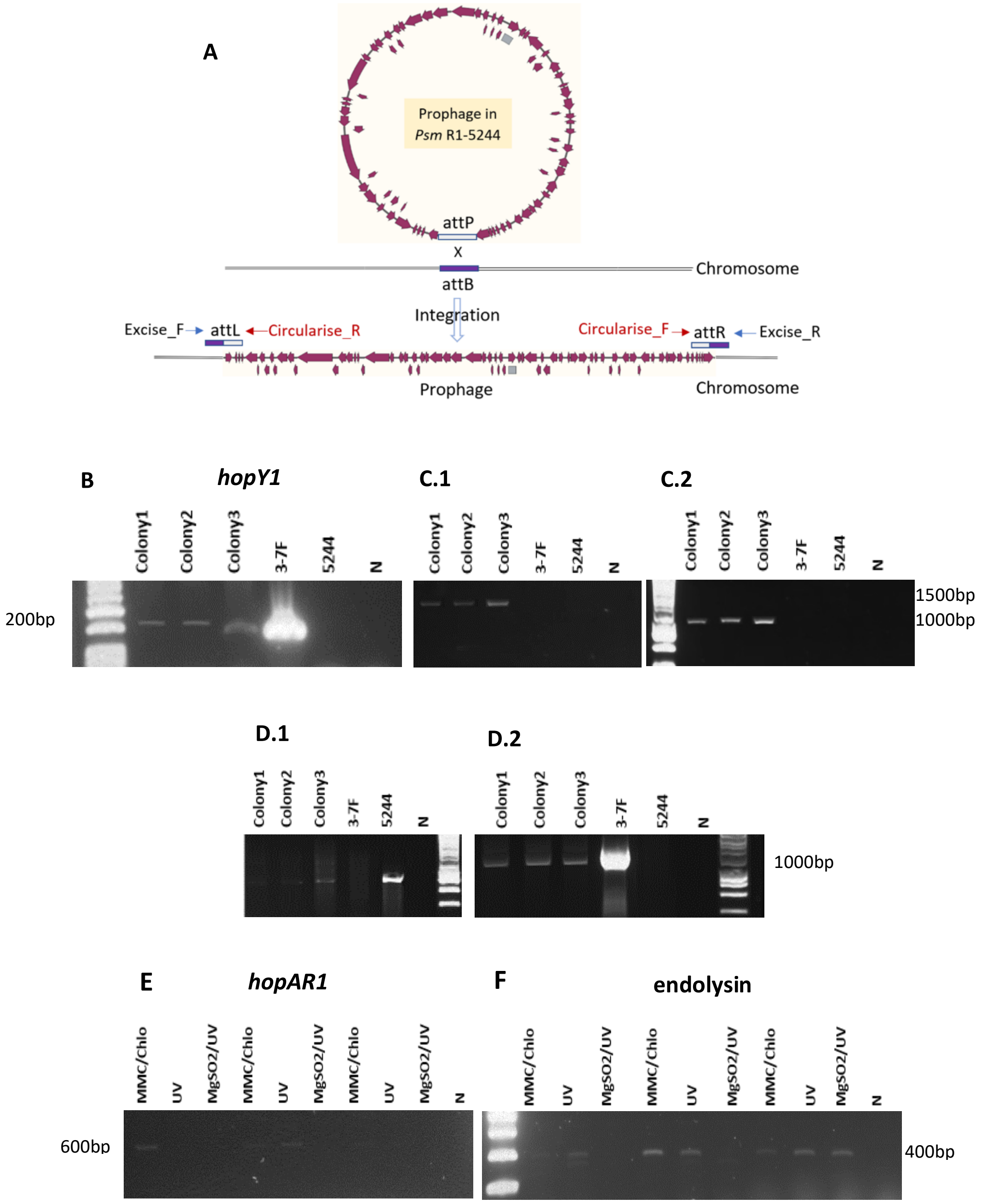

Figure S6. Transfer of the *hopAR1*-encoding prophage from *Psm* R1-5244_*Gm*^{R} into *P. syringae* phylogroup 10 strain 3-7F_*Rif* on cherry leaves following UV radiation. Excision, circularisation and induction of the prophage harbouring *hopAR1* from 3-7F_*Rif* was checked. A: Diagram of prophage integration into the recipient bacterial chromosome. Phage and bacterial DNA align at the attachment sites (*attP* and *attB*). The phage was integrated into the host chromosome bounded by attachment sites, *attL* and *attR*. B: Three Rif and *Gm* resistant colonies were checked by PCR for the *hopY1* gene which is present in *Ps* 3-7F_*Rif* and *Psm* R1 5244_*Gm*^{R} were used as control; C: Integration of prophage into the recipient strain was confirmed using different primer pairs in chromosomal DNA of the recipient. C.1: Excise_3-7F_F + Circularise_prophage_R, C.2: Circularise_prophage_F + Excise_3-7F_R. D: Excision and circularisation of phage from recipient strain was confirmed using different primer pairs in chromosomal DNA of recipient. D.1: Circularise_prophage_F + circularise_prophage_R and D.2: Excise_3-7F_F + Excise_3-7F_R. 1-3: recipient colonies

collected from plates supplemented with antibiotics. E: PCR performed for *hopAR1* and endolysin genes after prophage induction and purification from the recipient strain (3 colonies), following mitomycin C (MMC) and chloroform (Chlo), UV radiation, and $MgSO_4$ wash and UV radiation. N: no template control, sizing hyperladder 1kb and 50bp (Bioline, UK).

References

Leong SA, Ditta GS, Helinski DR. 1982. Heme biosynthesis in *Rhizobium*. Identification of a cloned gene coding for delta-aminolevulinic acid synthetase from *Rhizobium meliloti*. *J Biol Chem* **257**(15): 8724-8730.

Scott TA, Heine D, Qin Z, Wilkinson B. 2017. An L-threonine transaldolase is required for L-threo-β-hydroxy-α-amino acid assembly during obafluorin biosynthesis. *Nat Commun* **8**:15935.

Spilker T, Coenye T, Vandamme P, LiPuma JJ. 2004. PCR-based assay for differentiation of *Pseudomonas aeruginosa*from other *Pseudomonas* species recovered from cystic fibrosis patients*. J Clin Microbiol* **42**(5):2074-2079.

Yamamoto, S, Kasai, H, Arnold, DA, Jackson, RW, Vivian, A, Harayama, S 2000. Phylogeny of the genus *Pseudomonas*: intrageneric structure reconstructed from the nucleotide sequences of *gyrB* and *rpoD* genes. *Microbiology.* **146**(10):2385-2394
